# Dual binding mode of “bitter sugars” to their human bitter taste receptor target

**DOI:** 10.1038/s41598-019-44805-z

**Published:** 2019-06-11

**Authors:** Fabrizio Fierro, Alejandro Giorgetti, Paolo Carloni, Wolfgang Meyerhof, Mercedes Alfonso-Prieto

**Affiliations:** 1 Computational Biomedicine, Institute for Advanced Simulation IAS-5 and Institute of Neuroscience and Medicine INM-9, Forschungszentrum Jülich, Jülich, Germany; 2 Department of Biology, Rheinisch-Westfälische Technische Hochschule Aachen, Aachen, Germany; 3 Department of Biotechnology, University of Verona, Verona, Italy; 4 JARA–HPC, IAS-5/INM-9 Computational Biomedicine, Forschungszentrum Jülich GmbH, Jülich, 52425 Germany; 5 Department of Physics, Rheinisch-Westfälische Technische Hochschule Aachen, Aachen, Germany; 6 VNU Key Laboratory “Multiscale Simulation of Complex Systems”, VNU University of Science, Vietnam National University, Hanoi, Vietnam; 7 Center for Integrative Physiology and Molecular Medicine (CIPMM), Saarland University, Homburg, Germany; 8 Cécile and Oskar Vogt Institute for Brain Research, Medical Faculty, Heinrich Heine University Düsseldorf, Düsseldorf, Germany

**Keywords:** Computational biophysics, Protein structure predictions, Molecular modelling, G protein-coupled receptors, Sensory processing

## Abstract

The 25 human bitter taste receptors (hTAS2Rs) are responsible for detecting bitter molecules present in food, and they also play several physiological and pathological roles in extraoral compartments. Therefore, understanding their ligand specificity is important both for food research and for pharmacological applications. Here we provide a molecular insight into the exquisite molecular recognition of bitter β-glycopyranosides by one of the members of this receptor subclass, hTAS2R16. Most of its agonists have in common the presence of a β-glycopyranose unit along with an extremely structurally diverse aglycon moiety. This poses the question of how hTAS2R16 can recognize such a large number of “bitter sugars”. By means of hybrid molecular mechanics/coarse grained molecular dynamics simulations, here we show that the three hTAS2R16 agonists salicin, arbutin and phenyl-β-D-glucopyranoside interact with the receptor through a previously unrecognized dual binding mode. Such mechanism may offer a seamless way to fit different aglycons inside the binding cavity, while maintaining the sugar bound, similar to the strategy used by several carbohydrate-binding lectins. Our prediction is validated a posteriori by comparison with mutagenesis data and also rationalizes a wealth of structure-activity relationship data. Therefore, our findings not only provide a deeper molecular characterization of the binding determinants for the three ligands studied here, but also give insights applicable to other hTAS2R16 agonists. Together with our results for other hTAS2Rs, this study paves the way to improve our overall understanding of the structural determinants of ligand specificity in bitter taste receptors.

## Introduction

Human bitter taste or taste type 2 receptors (hTAS2Rs) are a group of 25 proteins (see Supplementary Table S1), belonging to the G-protein coupled receptor (GPCR) superfamily, the largest group of membrane proteins in humans^1^. Their classification within the GPCR superfamily has been the subject of debate. Based on evolutionary analysis, they were initially considered as part of class F, with which they share several common features, such as IFL in transmembrane helix (TM) 2, SFLL in TM5, and SxKTL in TM7^2^. However, this classification was suggested with reservations, since more work may be required to clarify the evolution of bitter taste receptors. More recent studies suggest that hTAS2Rs belong to class A. Indeed, they share several conserved motifs (see references^3–5^). Nonetheless, the low sequence similarity with class A GPCRs also supports their classification as a distinct family (class T)^6^.

Originally, bitter taste receptors were identified in the taste buds of the tongue^7,8^, where they detect bitter molecules. These include several toxic compounds^9^, molecules with nutritional beneficial properties^10–13^ or that add a valued property to foods^14–17^. Nonetheless, in the last years hTAS2Rs have also been identified in extraoral tissues, including the brain^18–20^, the gut^21,22^ the upper and lower airways^23,24^ and the heart^25^. Depending on their location, they play different physiological roles and have been shown to be associated with different diseases^22,26,27^. Hence, hTAS2Rs constitute exciting novel targets for pharmaceutical intervention^26–29^. For instance, hTAS2R38 and hTAS2R14 are expressed in the upper airways and their activation is involved both in production of nitric oxide and in improvement of the cilia beat frequency, triggering antibacterial mechanisms and facilitating mucus excretion^30^. Another example is hTAS2R16, which is expressed in the brain and whose activation by salicin might modulate neurite outgrowth^20^.

In spite of their physiological and pathological importance, experimental structural information on hTAS2Rs is missing. Hence, molecular level understanding of ligand binding to hTAS2Rs currently relies on computational methods (namely homology modeling, docking and molecular dynamics (MD) simulations), combined with experimental mutagenesis and functional data^4,31^. Unfortunately, hTAS2Rs display a low sequence identity (<20%) with any of the available GPCR templates (62 unique GPCR structures, of which 52 correspond to class A, as of March 2019^6,32^). As a consequence, the resulting homology models have low resolution^33–35^, regardless of the template used. The three dimensional structure, particularly the orientation of the side chains, is likely to be not correct, affecting the accuracy of the docking in predicting receptor/ligand interactions. Hence, MD simulations can be used to refine these low resolution models^4,31^, in particular, the hybrid molecular mechanics/coarse grained (MM/CG) approach. This was tested using the β2-adrenergic receptor (β2AR), a class A GPCR for which the crystal structure is available^36^. A model of the receptor was built based on a template with sequence identity comparable to that of hTAS2R/GPCR and the ligand was introduced in the structure using molecular docking. After a 0.8 μs MM/CG simulation, the obtained binding pose turned out to reproduce the receptor/ligand interactions present in the crystal structure of β2AR, attesting the validity of this computational protocol^37^. Furthermore, this methodology has also been used to study ligand binding in GPR3, a class A GPCR^38^. In the case of hTAS2Rs, MM/CG simulations have suggested receptor/ligand interactions in agreement with the binding residues inferred from the experiments^37,39^. Moreover, the simulations predicted new binding residues (not previously suggested by the experimental data) that were successfully confirmed *a posteriori* by carrying out additional experiments. Altogether, these integrated computational and experimental studies have provided important insights on agonist selectivity in bitter taste receptors^37,39^, as previously demonstrated for other GPCRs^40^.

While most of hTAS2Rs feature an intermediate or a narrow agonist spectrum, two outlier groups can be singled out: the broadly-tuned and the group-selective receptors^41^. The first group, which includes hTAS2R10, hTAS2R14 and hTAS2R46, can recognize almost half of the ~100 agonists tested against the whole set of 25 hTAS2Rs^41–44^. A possible rationale for such large agonist diversity has been put forward based on computer-aided structural predictions of hTAS2R46. This receptor shows a transient binding site – other than the canonical, orthosteric one – that might filter the receptor agonists out of the pool of bitter tastants^39^. This “access control”^45^ is also present in other class A GPCRs^46–50^.

**Figure 1:**
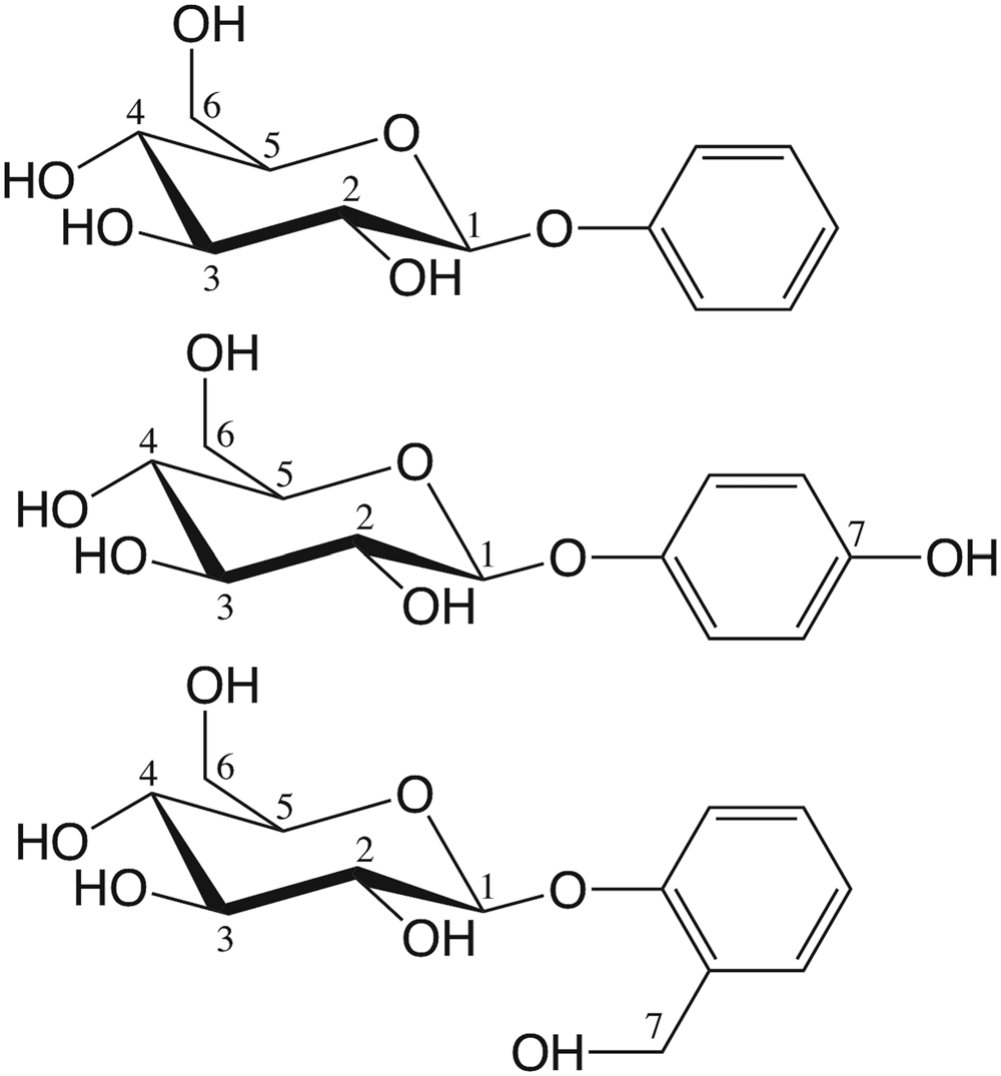
Chemical structures of the three agonists considered in this work: phenyl-β-D-glucopyranoside, arbutin and salicin (from top to bottom). Numbering of the glucopyranoside carbon atoms (and the corresponding oxygen atoms) is indicated; the phenyl substituent is numbered 7 for both arbutin and salicin for the sake of simplicity, despite the different position (*para* and *ortho*, respectively).

The hTAS2R46 agonist diversity contrasts sharply with the ligand selectivity of the group-selective receptors hTAS2R38 and hTAS2R16. The first is the target predominantly of bitter compounds containing an isothiocyanate or thiourea group^37,42,51^. In line with its high specificity, computer-aided predictions on hTAS2R38^37,51^ have not identified (as yet) any transient binding site possibly serving as “access control”, differently from hTAS2R46^31^. The other group-selective receptor, hTAS2R16, mainly recognizes bitter β-D-glycopyranosides (hereafter, “bitter sugars”)^52,53^. These are composed by a sugar (usually β-glucose, but also β-mannose in a few cases^52,53^) attached to a hydrophobic aglycon moiety; the latter can be extremely diverse (see Fig. 1 and Supplementary Fig. S1). Indeed, studies focused on the ligand selectivity of hTAS2R16 discovered about 30 diverse β-glycopyranosides agonists^53–58^, but this number could be even larger^42,59,60^ (see Supplementary Text S1). This poses the question of how hTAS2R16 can accommodate so many highly diverse ligands belonging to the same chemical class.

A structural characterization of the ligand binding determinants in hTAS2R16 would be crucial to understand the peculiar preference of hTAS2R16 for its agonists. Moreover, it may help design rationally potential novel drugs^61^, since several hTAS2R16 agonists are already known to affect human health^62,63^. These include arbutin, an inhibitor of bladder cancer proliferation^64^, and salicin, which has been long used for its analgesic, anti-inflammatory and antipyretic properties^65^. Salicin is a particularly interesting, yet challenging, lead compound, since it inhibits tumor growth and angiogenesis in endothelial cells^66^, but it can also have adverse effects by promoting neurite growth in neuroblastoma^20^.

Previous studies have attempted to characterize the structural determinants of agonist binding to hTAS2R16 (Supplementary Text S2). In an elegant series of experiments^55,56^, Sakurai and co-workers used site-directed mutagenesis to identify several residues that might be involved in agonist binding (Supplementary Table S2). They further showed that the mutations have similar effects across different ligands (such as salicin, arbutin and phenyl-β-D-glucopyranoside, see Fig. 1)^55,56^. This has led to the suggestion that all the receptor agonists share the same binding cavity^55^. In addition, Sakurai and coworkers proposed a structural model of the salicin binding pose^55^. However, this model does not predict ligand/receptor interactions with residues E262, L59 and V265, emerging from subsequent site-directed mutagenesis experiments^53^. This discrepancy may be consistent with the fact that bioinformatics-based models of human bitter taste receptors have only a limited predictive power^31^. Subsequently, Chen *et al*.^67^ presented a second model of the receptor in complex with salicin, using a computational approach similar to Sakurai, followed by molecular dynamics. Unfortunately, also this prediction seems not to be consistent with all the available mutagenesis data. In particular, residues F93, F236, F240, I243 and V265, putatively involved in ligand binding^53,55^ do not appear to interact with the ligand in their model. Therefore, none of the previously proposed models seems to be fully compatible with the available experimental data^53,55^ (see Supplementary Text S2 for details).

Classical MD simulations for low resolution models of chemosensory GPCR complexes appear to be not optimal because of TM stability issues. Indeed, the initial wrong orientation of the side chains may cause TM unfolding during the simulations. Hence, in an effort at shedding light on hTAS2R16/agonist interactions, here we use homology modeling and docking followed by extensive multiscale molecular mechanics/coarse grained (MM/CG) simulations^68–71^ on this receptor. The MM/CG approach has been shown to dramatically improve the predictive power of the computational models compared to using only bioinformatics methods^31^, by enhancing the sampling of the ligand binding cavity. Furthermore, the use of a Go-like model for the CG region helps to reduce the unfolding artifacts in the intracellular part of the receptor due to the low resolution of the initial model. Here we focus on three hTAS2R16 agonists: salicin, arbutin, and phenyl-β-D-glucopyranoside^53,55,56^. Our simulations (Supplementary Table S3) are validated *a posteriori* by comparison with available experimental site-directed mutagenesis data. Moreover they suggest that these three ligands display two possible binding modes for hTAS2R16, both consistent with the experimental data^53,55,56^. We hypothesize that this previously unrecognized dual binding mode mechanism might allow the receptor to accommodate hydrophobic aglycons of disparate sizes and with different substituents, thus helping hTAS2R16 to recognize a wider spectrum of bitter sugars. Based on these results, we also provide insights into the binding determinants of other hTAS2R16 agonists for which structure-activity relationship data are available.

## Methods

### Homology modelling

The sequences of the 25 hTAS2Rs were retrieved from the Pfam database^72^. The multiple sequence alignment (MSA) was generated using PROMALS^73^ and its correctness was checked by ensuring the alignment of conserved X.50 positions^32,74^ and conserved structural motifs across hTAS2Rs^5^. This MSA was used as input for the GOMoDo webserver^75^. GOMoDo uses HHsearch 2.0.16^76,77^ to convert the input MSA into a Hidden Markov Model (HMM) and then aligns this HMM to the HMMs of all the possible GPCR templates available in the GOMoDo database. A two step procedure was used for template selection and model building. In the first step, the target-template HMM alignments were used to build only 10 models of hTAS2R16 (UniprotKB ID: Q9NYV7) for each of the templates stored in the GOMoDo webserver (https://gomodo.grs.kfa-juelich.de/) using the MODELLER program (version 9.10)^78^ included in GOMoDo. The selection of the most suitable template was performed using both sequence and structural parameters: (i) we discarded models based on inactive templates, as agonists bind to the active state of the receptor and no significantly higher sequence identity with inactive templates was detected^79^; (ii) we further discarded models generated using template-target alignments with gaps inside the TM regions longer than one residue; and (iii) we selected models with higher GA341 scores^80^ and lower DOPE scores^81^, i.e. higher overall structural quality. The selected template turned out to be the β2 adrenergic receptor (β2AR, PDB ID 4LDE, sequence identity 13%). The alignment between hTAS2R16 and β2AR was visually inspected to ensure that the conserved features shared between hTAS2Rs and class A GPCRs were well aligned^5^.

Once the β2AR template was selected, the second modeling step was performed. Using the aforementioned target-template alignment (Supplementary Fig. S2), 200 new models were generated using a standalone version of MODELLER (version 9.11). This second step is intended to have more solutions during the model building and optimization step at a reasonable computational cost, since the standalone version of MODELLER is faster than the implementation in the GOMoDo webserver. The most suitable β2AR-based model among the 200 was selected using the following structural criteria: (i) high quality scores, (ii) preservation of the secondary structure of the TM helices and (iii) lower number of residues in not allowed regions of the Ramachandran plot. The protonation state of the titratable residues was determined by the hydrogen placement algorithm in MolProbity, which optimizes hydrogen bonding networks and minimizes steric clashes^82^.

### Ligand docking

The chemical structures of the ligands in Fig. 1 were downloaded from the PubChem database^83^. Their protonation states were identified using the pKa plugin in MarvinSketch (http://www.chemaxon.com/products/marvin/marvinsketch/). All three ligands have zero total charge.

Docking of the phenyl-β-D-glucopyranoside ligand to the hTAS2R16 model was performed with the HADDOCK webserver^84,85^. Fpocket^86,87^ was used to predict receptor residues putatively involved in ligand binding. After discarding residues belonging to the extracellular loops or pointing outside the receptor cavity, the remaining fpocket-predicted residues were used as active residues to define the so-called ambiguous interaction restraints (AIRs) for the data-driven HADDOCK procedure^84,85^. First, 1,000 initial structures were generated by rigid body docking and the 200 structures with lowest HADDOCK scores were submitted to further refinement using semi-flexible simulated annealing, followed by flexible explicit solvent refinement with a water layer. The resulting complexes were clustered using the algorithm in reference^88^ and a root mean square deviation (RMSD) cutoff of 2 Å. Clustering was used to compensate (at least in part) the known limitations of docking scores in ranking docking poses.

Indeed, the binding pose selected was the one with the lowest HADDOCK score belonging to the most populated cluster. In addition, another binding pose was also manually built using the tools of VMD^89^ (further details in the Results section). We would also like to note here the limitations of docking scores in discriminating the best docking poses for low resolution GPCR models^90–94^.

Since arbutin and salicin only differ by a small phenyl substituent, it is reasonable to assume that they are also accommodated in the same binding pocket and with the same orientation as phenyl-β-D-glucopyranoside. Consistently, mutations of residues presumably involved in binding affect similarly all three agonists^53,55,56^. Hence, a snapshot of the MD simulation of phenyl-β-D-glucopyranoside was taken at 12.5 ns (when the ligand is already located in the orthosteric binding site) and the phenyl aglycon was alchemically modified by adding the corresponding substituent (hydroxyl group *in para* for arbutin or hydroxymethyl group *in ortho* for salicin) with the Molefacture plugin in VMD^89^. Based on the second binding pose built for phenyl-β-D-glucopyranoside, a second binding pose was also manually built for arbutin and salicin using VMD^89^ (see Results).

### Multiscale molecular dynamics simulations

**Figure 2:**
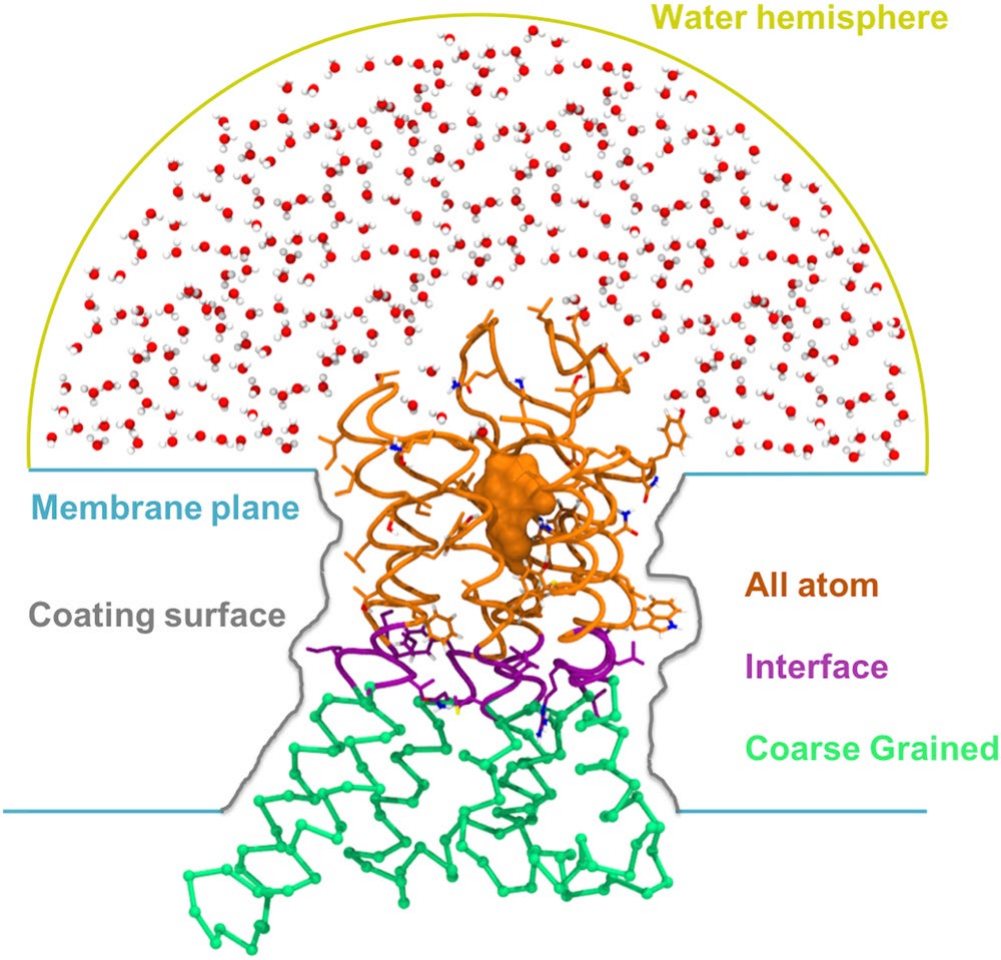
Molecular mechanics/coarse grained (MM/CG) simulation setup. Water molecules, the extracellular part of the receptor and the ligand (in orange) constitute the all atom (MM) region, whereas the intracellular part of the receptor (in green) is the CG region. The interface (in violet) mediates the interaction between the MM and the CG regions. Carbon atoms in the atomistic and in the interface regions are coloured according to the region they belong to, whereas hydrogen atoms are in white, nitrogen in blue and oxygen in red. The potentials used to mimic the presence of the membrane and to cap the water molecules are represented as colored lines (blue, gray and yellow, respectively).

In order to improve the bioinformatics predictions, the hTAS2R16 complexes were refined by performing molecular dynamics simulations, using the molecular mechanics/coarse grained (MM/CG) approach^68–70^. Figure 2 shows the simulation setup. The MM part consists of the extracellular part of the receptor, including the binding site with the docked ligand, along with the solvent (approx. 5,300 water molecules). The protein was described using the GROMOS96 43a1 force field^95^, whereas the SPC model was used for the water molecules^96^. The parameters for the ligands were built by combining GROMOS 56a6_CARBO forcefield^97^ parameters for the β-D-glucopyranose unit and PRODRG^98^ generated parameters for the aglycons. Following reference^99^, RESP charges^100^ were used for all ligands, calculated with Gaussian09^101^ at the B3LYP/6-31G(d,p) level of theory and fitted with Antechamber^102^. The intracellular part of the receptor, which is not involved in binding, was defined as the CG region and described using a Go-like potential^103^ between CG beads centered on the C_α_ atoms of the corresponding residues. We would like to note here that only the CG part is constrained (using the Go-like potential), whereas the MM part is fully flexible, both at the backbone and the side chain levels. In the MM/CG approach, the MM and the CG parts are connected by an interface region, which mediates the bonded and non-bonded interactions between the two levels of resolution. The membrane is represented in an implicit way, by introducing wall potentials (indicated with lines in Fig. 2). Further details can be found in recent reviews^104–106^.

Six hTAS2R16 complexes were considered (three agonists, two binding poses for each). Each complex was first energy minimized with three runs of steepest descent followed by three runs of conjugate gradient minimization. In each minimization run performed with the same algorithm, the maximum minimization step size was progressively increased by one order of magnitude (from 0.001 to 0.01 to 0.1 nm). Each minimization was carried out until no further changes in potential energy were detected or the maximum force was smaller than the threshold value of 10 kJ mol^−1^ nm^−1^. Next, a simulated annealing protocol was carried out to increase the temperature of the complex from 0 to 300 K in 6 ns, using a velocity rescaling thermostat. During the heating, positional restraints were applied on the protein backbone using a force constant of 1000 kJ/mol*nm^2^ in each direction. After the simulated annealing phase, three further equilibration steps of 3 ns each were performed. In each step, the force constant applied on the protein backbone was progressively decreased (from 500 to 250 to 125 kJ/mol*nm^2^). After removing all restraints, two simulations were run for each ligand, starting from two different binding poses (Supplementary Table S3). All simulations were run for 0.8 μs using a timestep of 2 fs.

### Interaction analysis

Hydrophobic interactions were calculated with the g_mindist tool of GROMACS^107^ and using a 5.5 Å distance cutoff (i.e., the sum of the van der Waals radii of two carbon atoms plus the diameter of a water molecule between them^108–110^). Hydrogen bonds (H-bonds) were identified with the “measure hbonds” tool in VMD^89^, using a 3.5 Å distance cutoff between donor and acceptor and a 30° deviation for the angle made by donor, hydrogen and acceptor atoms. The same criteria were used to define water-mediated H-bonds, in which the same water molecule is interacting simultaneously with both receptor and ligand. The persistency of a given interaction was calculated as the number of frames showing this interaction over the total number of frames, after excluding the first 100 ns of the simulation. Residues with hydrogen bond persistency higher than 10% or hydrophobic interaction persistency higher than 80% were considered as “computational binding” residues. We used two different persistency cutoffs for the two types of interactions to account for the higher fluctuations of the hydrogen bond network compared to the hydrophobic interactions. The rest were defined as “computational non-binding” residues. For the analysis of the static docking poses the computational binding and non binding residues were defined differently from the analysis of the MD trajectories. Indeed, they were identified based on the presence/absence of the interaction (defined as explained above) in the corresponding docked complex, as done in reference^31^. In other words, we excluded the persistency criterium because the interactions in the static binding poses are not time dependent, since no MD simulation had been run at this point.

A representative snapshot was selected for each simulation as follows. We first calculated the average structure with the GROMACS g_covar tool^111^ considering the whole system (except for the water molecules). Then, we computed the RMSD along the simulation using the GROMACS g_rms tool^111^, including only the transmembrane helices and the ligand. The snapshot with the lowest RMSD compared to the average structure was chosen as the representative structure of the corresponding ligand binding mode. The associated images were generated with VMD^89^.

Based on site-directed mutagenesis data and functional assays (agonist dose-response curves) data^53,55^, we also defined “experimental binding” and “experimental non binding” residues. In principle, changes in the half maximal effective concentration (EC_50_) values between the mutated and the wild-type (wt) receptor should be characteristic of residues involved in agonist binding, while an effect on the amplitude of the dose response curve upon mutation should be related with receptor activation^112,113^. However, in practice, it is difficult to ascertain whether a residue is involved only in binding, only in activation or in both. Actually, conformation-dependent effects^114^, changes indirectly related to the ligand affinity of the receptor (i.e. shaping of the binding cavity^37,39^) and second-shell effects^115^ can also affect the EC_50_ changes. Therefore, we used a second criterium based on the crystallographic structures of class A GPCR complexes^79^. Out of the residues whose mutation affects the EC_50_ value, only those located in the upper extracellular part of the receptor (i.e. the location of the canonical orthosteric binding site in class A GPCRs^79^) were considered as “experimental binding” residues, as done in reference^31^. Residues whose mutation does not change EC_50_ and/or that are located in the lower intracellular part of the receptor were considered as “experimental non-binding” residues.

Comparison of the computational and experimental residues allows us to define four different groups (Supplementary Text S3). “True positives” (TP) are amino acids identified as binding residues by both experiment and computation; “false positives” (FP) are amino acids identified as non-binding residues by experiment, but as binding residues in computation; “true negatives” (TN) are amino acids identified as non-binding residues by both experiment and computation; and “false negatives” (FN) are amino acids identified as binding residues by experiment, but not in computation. These were used to calculate the statistical parameters precision (PREC) and recall (REC):

$${\rm{PREC}}={\rm{TP}}/({\rm{TP}}+{\rm{FP}})$$

$${\rm{REC}}={\rm{TP}}/({\rm{TP}}+{\rm{FN}})$$

in order to assess the agreement of the computational models with the experimental data, as done in our previous work^31^. These are two statistical parameters commonly used to evaluate method performance^116–119^. They are close to 1 when the computational predictions are consistent with the experimental data, and zero when they are not. Precision evaluates how many residues are correctly predicted as important for ligand-receptor interactions, whereas recall quantifies how many of the experimental binding residues are captured by the simulations. Precision and recall values were calculated for the initial docking poses and for a representative snapshot of each simulation. We did not consider other statistical parameters including TNs because mutagenesis experiments are designed in order to capture binding residues^55^ and hence the number of potential TNs is very low (e.g. one out of seven for hTAS2R16).

## Results and Discussion

### Phenyl-β-D-glucopyranoside

As in our previous MM/CG investigations of hTAS2Rs^37,39^, we started from the binding pose obtained by homology modelling and molecular docking. In this pose, the ligand is almost parallel to the receptor axis, with the aglycon moiety buried inside the receptor and the glucose unit pointing towards the extracellular side. The reliability of this initial docking pose was assessed by comparison with the available experimental mutagenesis data, in terms of precision and recall. The values of these two statistical parameters are equal to 0 (Table 1 and Supplementary Table S2), indicating a low agreement of the initial docking pose with the experimental data. Although the fpocket predicted residues used to drive the docking included some of the binding residues inferred from the mutagenesis data, the docking pose is not accurate enough, most likely due to the wrong orientation of the residue side chains in the low resolution homology model.

**Table 1:** Precision and recall values for the computational models of the hTAS2R16 complexes.

	PGP dock	PGP TM3	PGP TM7	ARB TM3	ARB TM7	SAL TM3	SAL TM7
Precision^a^	0.0	0.83	0.83	0.80	0.83	1.00	1.00
Recall^b^	0.0	0.83	0.83	0.66	0.83	0.66	0.66

Three different ligands are considered (PGP, phenyl-β-D-glucopyranoside; ARB, arbutin; and SAL, salicin), each in two possible binding modes. For PGP, the values of the initial docking pose are also included.

^a^The precision of some complexes is lower than 1 because Q177, an experimental non-binding residue (see Supplementary Table S2), interacts with the ligand in some of the simulations and thus it is classified as FP.

^b^None of the complexes reached a recall value equal to 1 because F240 and/or to I243, experimental binding residues, do not interact with the ligand in some of the simulations and thus they are classified as FNs. Nonetheless, a molecular explanation of their roles can still be suggested (see text).

Hence, the docking pose was refined by performing MM/CG simulations. After a few ns, the phenyl-β-D-glucopyranoside ligand moved further inside the receptor and stabilized in this position for the rest (~0.8 μs) of the simulation (Supplementary Fig. S3). The identified binding cavity overlaps with the canonical orthosteric site observed in X-ray structures of class A GPCR/ligand complexes^79^ (Supplementary Fig. S4) and the receptor residues shown to be involved in binding are in agreement with the mutagenesis data (see below).

**Figure 3:**
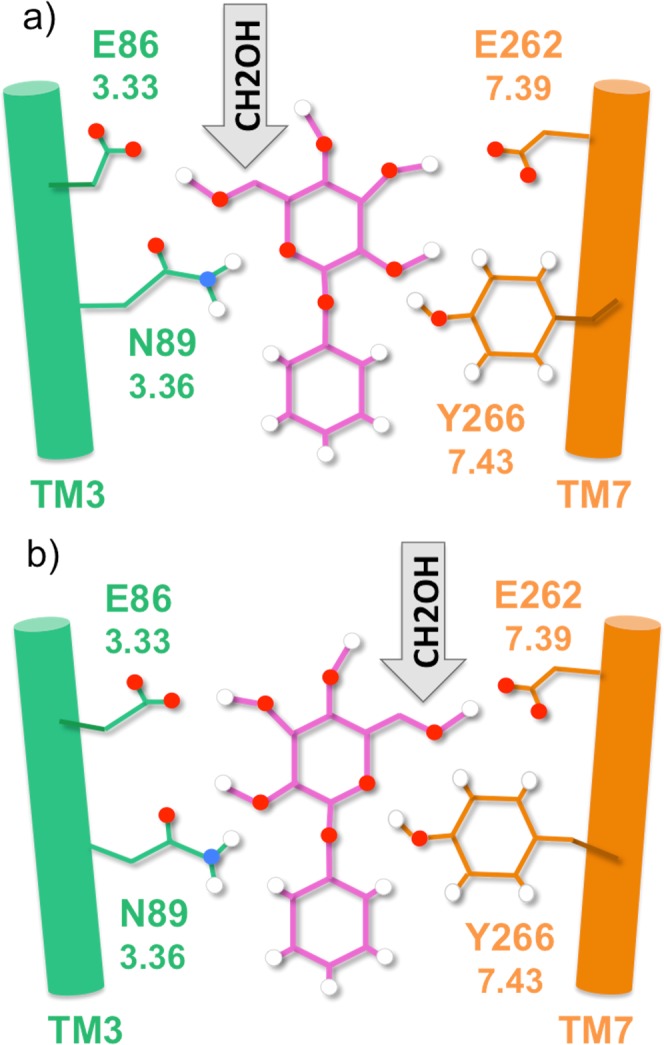
Some of the residues interacting through H-bonds with phenyl-β-D-glucopyranoside. The ligand is shown with pink lines, whereas residues on TM3 are represented with green lines and the counterparts on TM7 with orange lines. Oxygen, hydrogen and nitrogen atoms are shown as red, white and blue spheres. The generalized Ballesteros-Weinstein numbering across class A GPCRs is reported below each residue (e.g. 3.33 for E86). (**a**) At the entrance of the binding cavity, residue E86 on TM3 mirrors E262 on TM7, as respectively N89 does with Y266 one helix turn below. They all form H-bonds with the ligand during the simulation, even if the H-bond pattern is dynamic. This binding mode was defined as TM3-facing, due to the orientation of the glucose hydroxymethyl group toward TM3. (**b**) Same as panel (a) but with the ligand rotated by 180 degrees along the receptor axis. The hydroxymethyl group is pointing toward the TM7, i.e. the TM7-facing binding mode.

**Figure 4:**
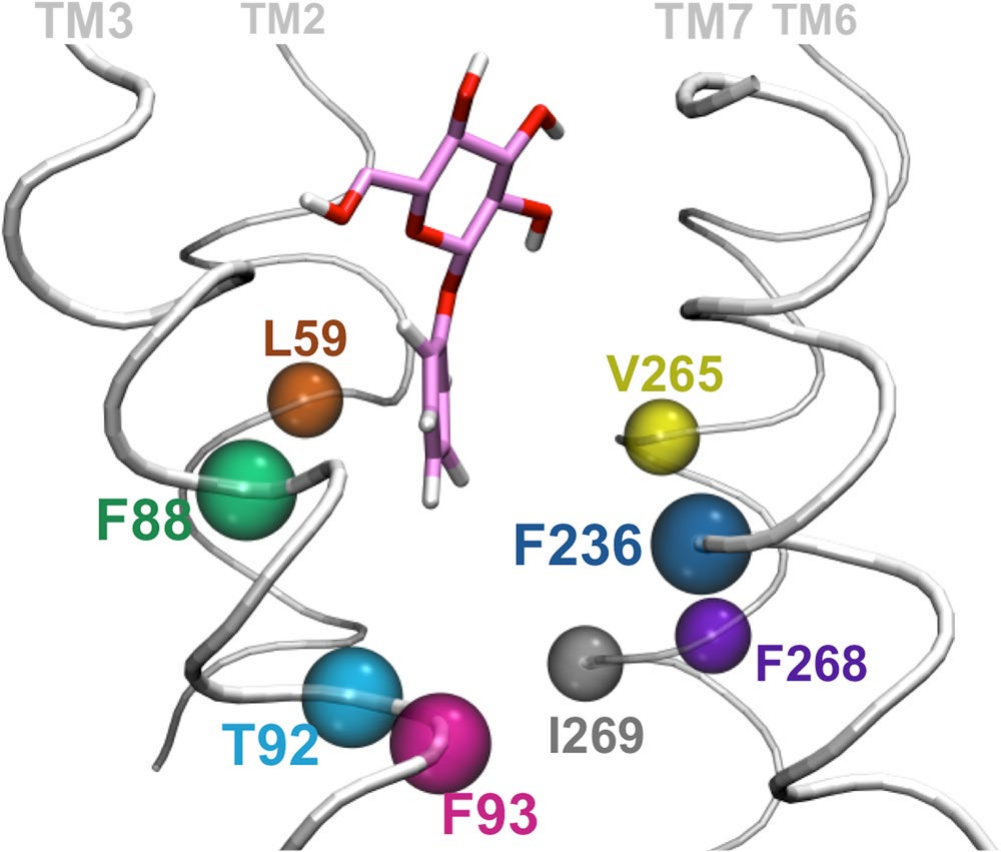
Main amino acids interacting with the ligand aglycon. Residues shaping the bottom part of the binding cavity are represented as spheres using different colors. The only polar residue among this set of aminoacid is T92.

In this binding pose, the glucose unit of the ligand is in contact with several polar residues in the upper part of the binding pocket (see below), as well as solvated by water molecules, while the aglycon hydrophobic moiety is mostly surrounded by hydrophobic residues in the bottom part. Therefore, the residue distribution of the receptor binding site appears to match the chemical properties of the sugar derivative. The glucose ring is held in place by hydrogen bonds (H-bonds) formed mainly with four residues, located in two adjacent transmembrane helices. These are E86^3.33^ and N89^3.36^ in TM3, and E262^7.39^ and Y266^7.43^ in TM7 (the Ballesteros-Weinstein numbering^74^ commonly used for class A GPCRs is indicated as superscript). These two sets of residues are mirroring each other, so that E86^3.33^ is in front of E262^7.39^, and similarly for the couple N89^3.36^ and Y266^7.43^ (Fig. 3). On the other hand, the phenyl moiety is surrounded almost completely by hydrophobic residues (see Fig. 4).

Given (i) the complementarity of the two sides of the binding cavity and (ii) the presence of hydroxyl groups on the ligand glucose moiety potentially able to form H-bonds with both sides of the previously described binding cavity, we asked ourselves whether a 180 degrees rotation of the phenyl-β-D-glucopyranoside along the receptor axis would still allow binding. The initial wrong orientation of the side chains and the limited exploration of the conformational space of the bioinformatics methodology may explain why this alternative binding pose was not identified by the docking algorithm. A further argument in support of the existence of a dual binding mode is the presence of a similar mechanism in several lectins^120–124^. In these sugar-binding proteins, the two binding modes involve the same residues, but use a different interaction pattern with the ligand. Therefore, we considered not only one but two possible binding modes: one in which the C6 substituent of the glucose ring is oriented towards TM3 (hereafter, TM3-facing mode, Fig. 3A) and the horizontally flipped alternative, in which the same substituent is pointing to TM7 (i.e. TM7-facing mode, Fig. 3B).

These two binding modes were investigated by running 0.8 microsecond MM/CG simulations (Supplementary Table S3 and Supplementary Fig. S3). In both cases, the ligand is stable inside the binding cavity (Supplementary Fig. S5). In addition, the receptor residues involved in interactions with the ligand are in agreement with the experimental mutagenesis data for both binding modes, as shown by the high values of recall and precision (see Table 1, Supplementary Table S2 and Supplementary Text S3). Hence, our simulations indicate that the phenyl-β-D-glucopyranoside agonist may have a dual binding mode.

In order to validate the quality of our predictions, an additional simulation was run, differing from the previously described in that the glucose unit is buried inside the receptor (instead of pointing toward the extracellular side) and the aglycon is pointing toward the extracellular rather than toward the inner part of the receptor (hereafter, glucose-in binding pose) (see Supplementary Text S2). This binding pose was discarded because of the instability of the ligand, which moves out of the receptor after a few nanoseconds (ca. 20) of MM/CG simulation (Supplementary Fig. S6). This behavior can be easily explained considering that initially the glucose unit is surrounded by hydrophobic residues, whereas the hydrophobic aglycon is surrounded by polar residues. The chemical mismatch between the glucose-in oriented ligand and the side chains constituting the binding site results in the glucose unit moving towards the extracellular side.

### Arbutin and salicin

Next, we examined two derivatives of phenyl-β-D-glucopyranoside, arbutin and salicin (Fig. 1), which are also experimentally characterized agonists of hTAS2R16^53,55^. The former differs from phenyl-β-D-glucopyranoside by the presence of a hydroxyl group in *para* position, whereas in the latter the phenyl substituent is a hydroxymethyl group in *ortho*. The corresponding complexes were obtained by alchemical modification of phenyl-β-D-glucopyranoside, in either its TM3- or TM7-facing binding modes. During the 0.8 μs MM/CG simulations, both arbutin and salicin turned out to explore the same binding cavity as phenyl-β-D-glucopyranoside and to share similar protein-ligand interactions (Supplementary Tables S4-S7). This is consistent with the experimental observation that mutations of residues presumably involved in binding affect similarly all the ligands tested^53,55,56^. Moreover, the receptor-ligand interactions observed in the simulations are also compatible with experimental mutagenesis data^53,55^, as shown by the high values of precision (0.8–1.0) and recall (0.66–0.83) obtained for both binding modes (see Table 1 and Supplementary Table S2). Therefore, the dual binding mode proposed above for phenyl-β-D-glucopyranoside seems to apply also to arbutin and salicin. Indeed, six different MD trajectories (3 ligands, 2 binding poses for each) for a total of 4.8 μs are consistent with the existence of the dual binding mode for hTAS2R16 agonists.

**Figure 5:**
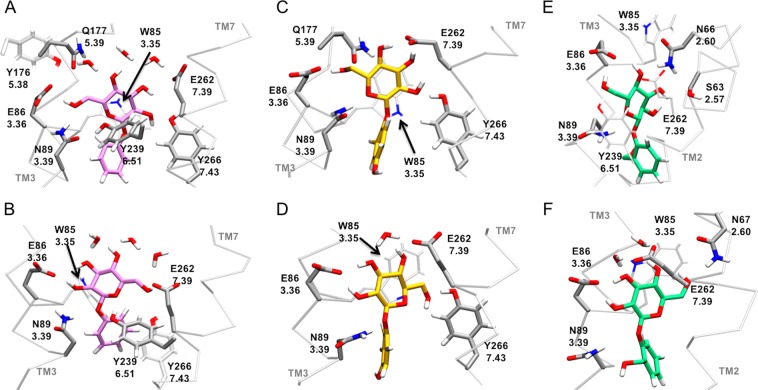
hTAS2R16 residues interacting with the glucose moiety of the ligands. Phenyl-β-D-glucopyranoside is shown as pink licorice (**A**,**B**), arbutin in yellow (**C**,**D**) and salicin in green (**E**,**F**). The top row (**A**,**C**,**E**) displays the TM3-binding mode of each ligand, while the TM7-binding mode is on the second row (**B**,**D**,**F**). The receptor interacting residues are in grey licorice and they are indicated with both their sequence and their Ballesteros-Weinstein numbering.

In the next sections, we will discuss the specific receptor-ligand interactions observed in the simulations (Fig. 5 and Supplementary Tables S4–S7), as well as the comparison with the available experimental data carried out to validate the proposed models (Supplementary Table S2).

### Molecular basis of β-glucopyranoside binding to hTAS2R16

#### Common interactions between hTAS2R16 and the glucose unit of the ligands *(W85*^*3*.*32*^, *E86*^*3*.*33*^, *N89*^*3*.*36*^, *E262*^*7*.*39*^*)*

Regardless of the ligand and the binding mode considered, the common glucose unit is mainly in contact with the same receptor residues (W85^3.32^, E86^3.33^, N89^3.36^, Y239^6.51^ and E262^7.39^), thus supporting the presence of a dual binding mode. However, the pattern of the interactions and their persistence varies among different complexes, as described below.

W85^3.32^ can form stacking or hydrophobic interactions with the glucose ring of the ligands (Fig. 5). This is in line with its mutation into R reducing the maximum response of the receptor towards salicin^53^, as well as the experimental data on this conserved tryptophan for other bitter taste receptors (reviewed in references^31,39^).

The two glutamates E86^3.33^ and E262^7.39^ are located in the upper part of the binding cavity and, in our simulations, they form H-bonds with the glucose hydroxyl groups in both binding modes (Fig. 5). In the TM3-facing mode (Fig. 5A,C,E and Supplementary Table S4), E86^3.33^ forms H-bonds mainly with O6, whereas in the TM7-facing mode (Fig. 5B,D,F and Supplementary Table S5), it does so with O2 and O3 (see oxygen numbering in Fig. 1). The persistency of these interactions along all the simulations suggests a fundamental role of E86^3.33^ in ligand binding, in agreement with the experimental data. In particular, the isosteric E86Q mutation almost does not modify the EC_50_ value for any of the three ligands (mutant/wild-type ratio between 2.9 and 5.7), while the E86D mutation has a stronger impact (between 7.9 and 18.4), indicating that the length of the side chain at position 3.33 is crucial to establish H-bonds with the glucose unit^55^. On the other hand, E262^7.39^ is more flexible than E86^3.33^, forming H-bonds with more than one glucose hydroxyl group and with variable persistency (Supplementary Tables S4 and S5). This is in line with the maximum receptor activity with salicin being only slightly decreased by the E262D mutation, compared to the stronger effect of E86D; only the E262A mutation completely abolishes receptor activation^53^. Moreover, mutations of the homologous E265^7.39^ in hTAS2R46 significantly altered the EC_50_ of this receptor for its cognate agonist^45^, thus further supporting the participation of position 7.39 in agonist binding in hTAS2Rs.

N89^3.36^ can act as a H-bond donor to O1 and/or O2 in the TM7-facing binding mode or to their symmetric counterparts O5 and/or O6 in the TM3-facing binding pose (Fig. 5). The role in ligand binding of N89^3.36^ is supported by the experimental data^53,55^, showing that its mutation abolishes the receptor activity for all three ligands.

Interestingly, residues W85^3.32^ and N89^3.36^ are highly conserved across the hTAS2R family (Supplementary Table S6) and mutagenesis data on other bitter taste receptors (see^31,39^ and references therein) also support their participation in the formation of the ligand binding cavity. In addition, residues at positions 3.32, 3.33, 3.36 and 7.39 discussed here, together with position 6.48 and 6.51 (see next sections), have been proposed to be part of the consensus ligand binding pocket across class A GPCRs^79^.

#### Common interactions between hTAS2R16 and the variable phenyl aglycon *of the ligands (L59*^*2*.*53*^, *F88*^*3*.*35*^, *T92*^*3*.*39*^, *F93*^*3*.*40*^, *F236*^*6*.*48*^, *V265*^*7*.*42*^, *F268*^*7*.*45*^, *I269*^*7*.*46*^*)*

The variable phenyl aglycon is surrounded mostly by hydrophobic and aromatic residues, including L59^2.53^, F88^3.35^, F93^3.40^, F236^6.48^, V265^7.42^, F268^7.45^ and I269^7.46^ (see Fig. 4); the only exception is the polar T92^3.39^. Out of these residues, mutagenesis data on hTAS2R16 are available for L59^2.53^, F236^6.48^ and V265^7.42^ (change in receptor response to salicin) and F93^3.40^ (change in EC_50_ for all three ligands)^53,55^. For salicin, mutation of three aforementioned residues reduce the receptor activity (to 64%, 2% and 5% of the wild-type, see Supplementary Table S2), whereas mutation of the latter decreased EC_50_ by 19 to 55-fold, depending on the mutation. Consistently, in our simulations they form hydrophobic interactions with the phenyl moiety of all three ligands and are also involved in shaping the binding cavity (Supplementary Tables S6 and S7).

In addition, our simulations indicate that F268^7.45^ interacts with all the agonists. Although there are no experimental data for this residue in hTAS2R16, position 7.45 is conserved among hTAS2Rs (Supplementary Table S8) and there are mutagenesis data available for other bitter taste receptors^31,39^ that support its participation in the formation of the ligand binding cavity. T92^3.39^ is also conserved across the whole bitter taste receptor family, but experimental data are missing. Nonetheless, position 3.39 is involved in sodium binding in class A GPCRs^125^ and thus we speculate that T92^3.39^ may also play a functional role in hTAS2Rs (see next section).

#### Differences in binding among the three ligands *(S63*^*2*.*57*^, *N67*^*2*.*61*^, *T92*^*3*.*39*^, *Y266*^*7*.*43*^*)*

As mentioned above, the common glucose unit establishes a stable, yet flexible, H-bond network with the receptor, which is very similar among the three ligands. The only significant differences involve salicin binding, most likely due to the bulkier hydroxymethyl substituent in *para* position of the phenyl aglycon. While the glucose moiety of arbutin and phenyl-β-D-glucopyranoside is mainly interacting with residues located on TM3 and TM7, salicin interacts with TM3 and TM2 residues. In other words, for salicin the TM2 helix helps TM7 to bind the ligand. In the TM3-facing binding mode, the O2 group of arbutin and phenyl-β-D-glucopyranoside is interacting with Y266^7.43^, while for salicin it does with S63^2.57^ and N66^2.60^ (Figs 4 and 5). In the TM7-facing mode, the O6 group of phenyl-β-D-glucopyranoside and arbutin also forms a H-bond with Y266^7.43^, whereas for salicin it is N67^2.61^. Unfortunately, no mutagenesis studies have been performed on S63^2.57^, N66^2.60^ and N67^2.61^, and thus their role in salicin binding remains to be verified. However, it is noteworthy that hTAS2R16 is the only bitter taste receptor having a H-bond donor/acceptor at position 2.57, whereas other hTAS2Rs have a hydrophobic residue (I or L, Supplementary Table S8). Therefore, it would be interesting to experimentally test the effect of mutations S63I and S63L on salicin binding.

T92^3.39^ is another residue interacting differently with the three ligands. Although the bottom part of the binding site is lined with hydrophobic and aromatic residues and thus it is likely to be specialized in binding the phenyl moiety common to all three ligands, this region also contains a single polar amino acid, T92^3.39^. This residue does not interact with phenyl-β-D-glucopyranoside, but its presence allows the formation of a H-bond with the ligands containing substituted phenyl aglycons, i.e. the phenolic oxygen of arbutin and the hydroxymethyl oxygen of salicin (O7 in Fig. 1). In the case of arbutin, a H-bond is present between the phenolic oxygen and T92^3.39^ for both binding poses, because the O7 atom in *para* is optimally positioned to point toward T92^3.39^. Instead, for salicin the H-bond pattern changes between the two binding modes, due to the O7 substituent being in *ortho* position. For the TM3-facing binding pose, the salicin hydroxymethyl group forms a H-bond with T92^3.39^, whereas, for the TM7-facing mode, salicin O7 can either form a weak interaction with T92^3.39^ or act as a H-bond acceptor to N89^3.36^. In other words, the salicin O7 substituent is always pointing towards TM3, regardless of the binding mode. Based on the results of our simulations, we predict that mutation of T92^3.39^ into a hydrophobic residue would affect the receptor response to salicin and arbutin, while the impact for phenyl-β-D-glucopyranoside might be weaker.

Moreover, position 3.39 is quite conserved across the hTAS2R family (see Supplementary Table S8), though there are no available mutagenesis data for any bitter taste receptor. Hence, we resorted to the information available for other class A GPCRs. In this class, it has been proposed that a conserved S/T at precisely this position forms part of the allosteric sodium binding site^125^. However, it should be noted that sodium binding in class A GPCRs requires a conserved D/E residue (located at position 2.50), and this acidic residue is replaced by a conserved R in hTAS2Rs. Therefore, one could surmise that the canonical sodium binding site might be missing in hTAS2Rs. Nonetheless, position 3.39 might still play a functional role in hTAS2Rs through a different mechanism, which remains to be tested with experiments.

#### Residues involved in shaping the binding cavity and in second shell effects (Q177^5.39^, H181^5.43^, Y239^6.51^, F240^6.52^, I243^6.55^)

The contact frequency analysis (Supplementary Tables S6 and S7) shows that, besides the aforementioned residues, H181^5.43^ is also close (i.e. within the 5.5 Å distance cutoff) for all three ligands. However, our simulations indicate that H181 is not directly interacting with the β-glucosides. Instead, it may have an indirect effect, since it forms a salt bridge with E86^3.33^ in all the simulations. This glutamate directly interacts with the ligand and its interaction with H181^5.43^ might keep E86^3.33^ in optimal position to act as H-bond acceptor for the glucose hydroxyl groups (Supplementary Fig. S7). In this regard, H181^5.43^ can be considered part of the second binding shell. Experimentally, the mutation H181T slightly reduces the EC_50_ with respect to wild-type hTAS2R16 for the three ligands (between 2 and 8.5 times), while H181L has a stronger impact on EC_50_ (no response for two out of the three ligands, see Supplementary Table S2)^55^. Therefore, the mutagenesis data supports the suggestion that a polar residue able to form H-bonds is needed at position 5.43.

Another second-shell residue is F240^6.52^. Although it is not directly interacting with many of the ligands, we noticed that it is part of an “aromatic cluster” which involves several π-stacked phenylalanines on TM6, TM7 and TM3. In particular, F240^6.52^ forms a T-stacking interaction with F236^6.48^, which in turn forms a parallel displaced stacking with F93^3.40^; the latter interacts with F268^7.45^ through parallel displaced stacking (Supplementary Fig. S8). Considering that F93^3.40^, F236^6.48^ and F268^7.45^ are involved in hydrophobic interactions with the ligand, F240^6.52^ may help to stabilize the other phenylalanines in the cluster and thus have a second shell effect in ligand binding. Mutation of F240^6.52^ to aromatic residues Y or W decreases EC_50_ only slightly (by 6.5–11-fold), whereas the L mutant shows no receptor activity^53,55^. Therefore, the experimental data supports that the stacking interactions of the aromatic cluster are likely to be important for ligand binding.

Besides, other residues possibly involved in shaping the binding cavity or in second shell effects according to our simulations are Q177^5.39^, Y239^6.51^ and I243^6.55^ (see Supplementary Tables S6 and S7). Q177^5.39^ is able to interact with the ligands through H-bonds (Supplementary Tables S4 and S5). However, the same hydroxyl group in contact with Q177^5.39^ can form more persistent H-bonds with other residues, suggesting that Q177^5.39^ is not essential for ligand binding or that can be easily replaced by other residues. Indeed, EC_50_ data shows that mutations to N, E or A do not affect the EC_50_ significantly (i.e. the EC_50_ change is only between 0.9 and 2.9 times, depending on the mutation and the ligand).

Y239^6.51^ can interact with the three ligands, but the low persistency of the corresponding H-bond (Supplementary Tables S4 and S5) is not enough to discriminate whether this residue is involved in ligand binding or in shaping the binding cavity. Indeed, in our simulations we observed that Y239^6.51^ can also establish a H-bond with N89^3.36^ (Fig. 5A), thus contributing to the correct orientation of the latter residue inside the binding pocket. Future mutations Y239F and Y239T could help to clarify the role of this residue.

Finally, I243^6.55^ is located one helix turn above Y239^6.51^. We predict that the presence of a bulky residue in position 6.55 might help to keep the aromatic ring of Y239^6.51^ close to the ligand. In other words, I243^6.55^ may help Y239^6.51^ to adopt the right rotameric state. This is in line with mutations to bulky hydrophobic residues (I243L and I243V) reducing only slightly the EC_50_ (mutant/wild-type ratio between 2.0 and 5.2), while mutation to A (a smaller residue) shows no receptor response for any of the ligands studied here^55,56^.

#### Overall picture

In summary, our calculations suggest that the upper part of the hTAS2R16 binding cavity is mostly composed by polar residues that interact with the glucose unit. Interestingly, the residues capable of forming H-bonds are positioned mirroring each other on both sides of the cavity (E86^3.33^ and N89^3.36^ on TM3, as well as E262^7.39^ and Y266^7.43^ on TM7). The only exception is represented by salicin, which replaces some of the interactions with residues located on TM7 by polar residues on TM2 (S63^2.57^, N66^2.60^ and N67^2.61^). Despite the slight differences for salicin binding, the picture remains the same for the three agonists studied here: the glucose ring is hold in place by two sets of polar residues, located in two spatially adjacent helices. In contrast, the bottom part of the hTAS2R16 binding cavity consists mostly of hydrophobic and aromatic residues that can interact with the hydrophobic aglycon (Fig. 4). Since these interacting residues are positioned all around the bottom part of the cavity and hydrophobic interactions are non-directional, the aglycon can be easily accommodated regardless of the ligand orientation (either TM3- or TM7-binding modes).

### Binding mode of other hTAS2R16 agonists

We next provide qualitative insights into the binding determinants of other bitter sugars for which experimental data are available^52–54^. Assuming that these other hTAS2R16 agonists adopt a binding pose similar to that of the three ligands studied here (arbutin, phenyl-β-D-glucopyranoside and salicin), one can hypothesize a rationale for the changes in receptor activity caused by the variation in chemical structure among the different agonists. As shown below, this structure-activity relationship (SAR) data gives further support to our simulations.

In addition to β-D-glucopyranosides, hTAS2R16 can also detect some disaccharides, such as amygdalin. The O6 addition of another glucose unit in amygdalin (Supplementary Fig. S1) does not affect the receptor response significantly^54^. This can be explained, at least in part, assuming that amygdalin adopts a binding pose with the glucose moiety pointing toward the extracellular side of the receptor, as the one described here. The additional sugar will be placed towards the solvent and might be able to interact with other residues in the upper part of the binding cavity.

Replacement of the phenyl aglycon by smaller aliphatic groups (such as in methyl- or hexyl-β-D-glucopyranoside) decreases the receptor response^53,54^. These SAR data also supports the orientation of the ligand proposed here, since the aforementioned substitutions would result in loss of hydrophobic interactions between the aglycon and residues in the bottom part of the binding cavity, compared to phenyl-β-D-glucopyranoside. Moreover, the lack of hTAS2R16 response to the β-glucose monosaccharide^52^ may be due to the removal of the hydrophobic aglycon otherwise present in hTAS2R16 agonists (Supplementary Fig. S1), which eliminates the stabilizing hydrophobic interactions with the bottom part of the binding cavity. This is also in line with the higher hydrophobicity of bitter tastants compared to sweet compounds^126^.

Besides the aglycon substitutions, it is also interesting to compare with the SAR data for the sugar unit. The β configuration of the glycosidic oxygen and the equatorial orientation of the 4-OH group (Fig. 1) are known to be essential for hTAS2R16 recognition^52–54^. Consistently, the O1 and O4 atoms of the three ligands studied here are both involved in H-bonds with the receptor. The lack of receptor response to α-D-glucopyranoside and β-D-galactopyranoside^52,54^ can thus be ascribed to the change in orientation of the glycosidic oxygen or the 4-OH group relative to β-glucopyranoside, respectively (Supplementary Fig. S1). This in turn may severely affect the H-bond network that keeps the ligand bound. In addition, hTAS2R16 does not respond to phenyl-β-D-xylopyranoside^53^, in which the glucose C6 hydroxymethyl group present in β-glucopyranoside is absent (Supplementary Fig. S1). This may be interpreted based on the loss of H-bond interactions (with E86^3.33^ and Y239^6.51^ in TM3-binding pose or with E262^7.39^ and Y266^7.43^ for TM7-binding pose).

In contrast, β-D-mannopyranoside (Supplementary Fig. S1) can still be detected by hTAS2R16^52,53^. This may be possible, at least in part, by an exchange of H-bonding residues upon epimerization of the 2-OH group from equatorial (glucose) to axial (mannose). In the TM3-facing mode, the O2 atom would go from forming H-bonds with E262^7.39^ and Y266^7.43^ for glucose to be near Y239^6.51^ for mannose. Complementarily, in the TM7-facing mode, W85^3.32^ could take the place of E86^3.33^ or N89^3.36^.

## Conclusions

We have presented here the binding determinants of hTAS2R16, a group-selective bitter taste receptor that preferentially binds β-glycopyranosides with a hydrophobic aglycon (i.e. bitter sugars). Our simulations suggest receptor-ligand interactions that are validated *a posteriori* by comparison with a plethora of experimental data. Moreover, they predict new putative binding residues not yet experimentally characterized, in particular S63^2.57^ and T92^3.39^. Additional mutagenesis and functional experiments, complemented with MD simulations of the mutated complexes, will help to clarify the putative role of these residues in binding^39,127,128^.

Although hTAS2R16 is highly specialized in detecting bitter β-glucopyranosides, its ligands display a large diversity of aglycons (Fig. 1 and Supplementary Fig. S1), varying in size and hydrophobicity^53–56^. This poses the question of how the binding cavity of hTAS2R16 is capable to adapt to such a broad range of ligands. Here, using MM/CG simulations on the complexes with the hTAS2R16 agonists arbutin, phenyl-β-D-glucopyranoside and salicin (Fig. 1), we suggest that the solution to this apparent dichotomy is the existence of a previously unrecognized dual binding mode. Unlike previously published models (see Supplementary Text S2 ^55,67^, our findings can provide a molecular explanation to all the available experimental data, even if the mutagenesis data were not used to drive the docking or bias the simulations. Moreover, they provide a rationale for the SAR data^53–56^ and thus give insights applicable to other hTAS2R16 agonists, besides the three studied here.

Our work suggests, for the first time, the presence of a dual binding mode mechanism in bitter taste receptors. Nonetheless, this is not the only protein exhibiting a dual binding mode. Several other protein-ligand complexes have been reported^129–146^, including several lectins^120–124^, i.e. carbohydrate-binding proteins that are able to recognize sugars with high specificity. In these lectin-sugar complexes, the two observed binding modes involve interactions between the same protein residues and the same sugar hydroxyl groups, but with a different H-bond pattern. This is essentially the same strategy used by hTAS2R16 to bind the glucose unit of the bitter sugar using both the TM3- and TM7- binding modes. In addition, for lectins it was shown that the two binding modes coexist in equilibrium, as observed in the crystal structures^120,121^, and can exchange at room temperature^122,123^. Although our simulations are not long enough to explore this exchange, we speculate that this might be also the case for the two binding modes identified here for the hTAS2R16 complexes.

The physico-chemical characteristics of the hTAS2R16/glycopyranoside complex are in agreement with the features suggested to be important to enable multiple binding modes in other protein-ligand complexes^136,138^. The first one is ligand flexibility, which allows the ligand to adopt several possible conformations with a low energy cost. For the bitter sugars considered here, the flexibility is ensured by the rotatable glycosidic bond, which permits to position the aglycon in different orientations. The second one is the presence of a mostly hydrophobic binding site, which can provide rather unspecific and non-directional interactions. In our case, the intracellular part of the hTAS2R16 binding cavity is surrounded by hydrophobic residues that can stabilize the hydrophobic aglycon of the bitter sugars regardless of the orientation. However, the extracellular part of the binding cavity is mostly composed by polar residues, in order to accommodate the glucose unit. hTAS2R16 overcomes this issue by placing two sets of interacting residues mirroring each other in adjacent helices (TM3 and TM7). This peculiar residue distribution is still compatible with the dual binding mode because it allows the formation of H-bonds with the sugar in either of the two 180 degree-rotated binding modes (see Supplementary Text S4).

The three hTAS2R16 agonists studied here (arbutin, phenyl-β-D-glucopyranoside and salicin) can bind to the receptor using either of the two binding modes. Nonetheless, given the large diversity of aglycons recognized by hTAS2R16, some might exhibit only one binding mode (specially those with non-symmetric aglycons and bulky substituents). Indeed, the changes in salicin binding compared to unsubstituted phenyl-β-D-glucopyranoside (Fig. 1) suggest that the dual binding mode of hTAS2R16 agonists might be modulated by the aglycon substituents. This is in line with a previous analysis of protein-ligand complexes exhibiting dual binding mode^136^ showing that two types of mechanisms can be observed. Ligands belonging to the same chemical class can bind either with two different orientations or with only one depending on their substituents.

In summary, our simulations suggest that the particular residue distribution of the hTAS2R16 binding cavity enables the existence of the dual binding mode mechanism, which in turn allows to accommodate a wide variety of ligands.

## Supplementary information

Supplementary Information revised

## Data Availability

Data generated or analyzed during the current study are available upon request.
