# Supplementary material for: Dual binding mode of “bitter sugars” to their human bitter taste receptor target
Source: Sci Rep. 2019 Jun 11;9:8437. doi: 10.1038/s41598-019-44805-z (PMC6560132; doi:10.1038/s41598-019-44805-z)
Supplement: Supplementary file 1 — Supplementary Information revised [file 41598_2019_44805_MOESM1_ESM.pdf]

Dual binding mode of "bitter sugars" to their human bitter taste receptor target

Supporting Material

Fabrizio Fierro, [1,2] Alejandro Giorgetti, [1,3,4] Paolo Carloni, [1,4,5,6] Wolfgang Meyerhof [7] and Mercedes Alfonso-Prieto*[1,4,8]

1. Computational Biomedicine, Institute for Advanced Simulation IAS-5 and Institute of Neuroscience and Medicine INM-9, Forschungszentrum Jülich, Jülich, Germany;
2. Department of Biology, Rheinisch-Westfälische Technische Hochschule Aachen, Aachen, Germany.
3. Department of Biotechnology, University of Verona, Verona, Italy.
4. JARA–HPC, IAS-5/INM-9 Computational Biomedicine, Forschungszentrum Jülich GmbH, Jülich 52425, Germany.
5. Department of Physics, Rheinisch-Westfälische Technische Hochschule Aachen, Aachen, Germany.
6. VNU Key Laboratory "Multiscale Simulation of Complex Systems", VNU University of Science, Vietnam National University, Hanoi, Vietnam.
7. Center for Integrative Physiology and Molecular Medicine (CIPMM), Saarland University Homburg, Germany.
8. Cécile and Oskar Vogt Institute for Brain Research, Medical Faculty, Heinrich Heine University Düsseldorf, Düsseldorf, Germany.

* Corresponding author; email: m.alfonso-prieto@fz-juelich.de

Table S1. List of hTAS2Rs proteins and additional name (from reference [1]).

Receptor name	Aliases
hTAS2R1	TRB7
hTAS2R3	/
hTAS2R4	/
hTAS2R5	/
hTAS2R7	TRB4
hTAS2R8	TRB5
hTAS2R9	TRB6
hTAS2R10	TRB2
hTAS2R13	TRB3
hTAS2R14	TRB1
hTAS2R16	/
hTAS2R38	hTAS2R61
hTAS2R39	hTAS2R57

Receptor name	Aliases
hTAS2R40	hTAS2R58, GPR60
hTAS2R41	hTAS2R59
hTAS2R42	hTAS2R55
hTAS2R43	hTAS2R52
hTAS2R44	hTAS2R31, hTAS2R53
hTAS2R45	GPR59
hTAS2R46	hTAS2R54
hTAS2R47	hTAS2R30
hTAS2R48	hTAS2R19, hTAS2R23
hTAS2R49	hTAS2R20, hTAS2R56
hTAS2R50	hTAS2R51
hTAS2R60	hTAS2R56

The nomenclature of the 25 human bitter taste receptors is far from being trivial, as discussed in references [2,3]. hTAS2Rs are not numbered consecutively because of the presence of pseudogenes and because of the confusion generated by the use of different names assigned to the same receptor when different research groups published their data almost at the same time [2,3]. For the hTAS2Rs discussed in our manuscript, we refer to the nomenclature in reference [4]. Nonetheless, hTAS2R16 is one of the few examples for which there are no aliases and the same name has been consistently used in the literature.

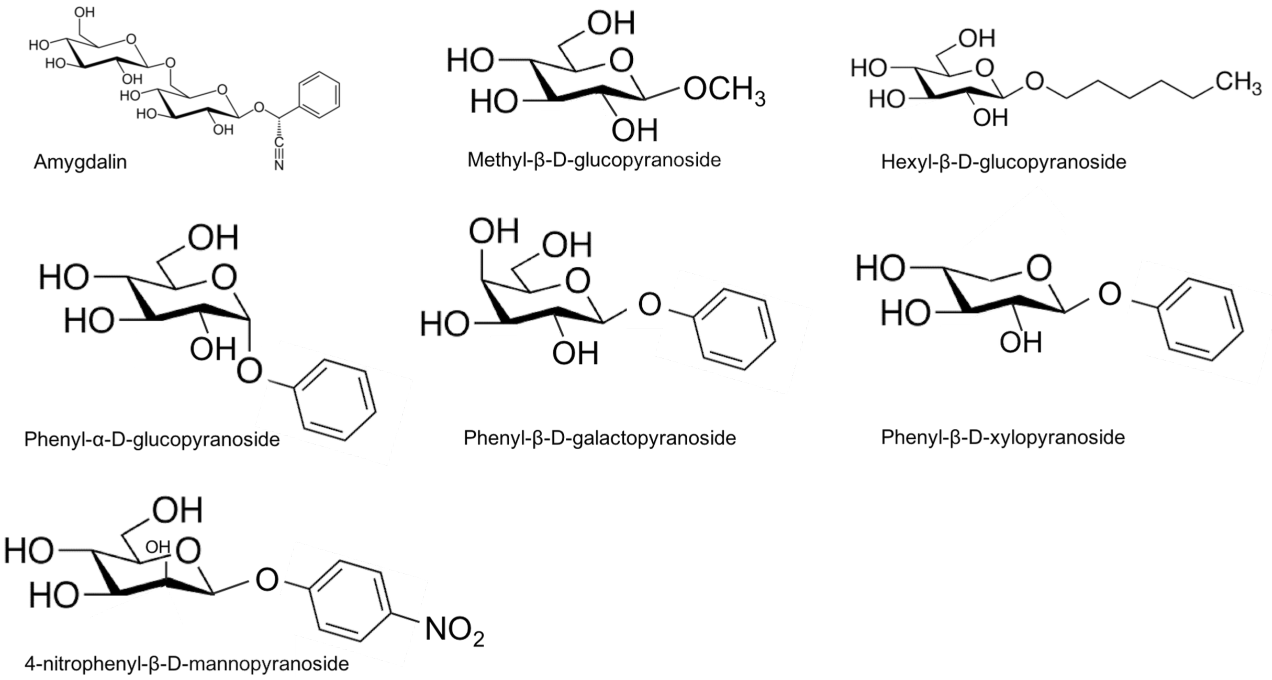

Figure S1. Chemical structure of the glycopyranosides mentioned in the main text.

Text S1. hTAS2Rs and hTAS2R16 tested compounds.

hTAS2R16 is one of the first human bitter taste receptors deorphanized [4], showing a high specificity for salicin and related bitter β-D-glycopyranosides (i.e. bitter sugars).

Meyerhof and coworkers [5] used a panel of 100 structurally diverse bitter molecules and tested whether they activate one or more of the 25 hTAS2Rs. Five out of these 100 compounds (salicin, helicin, amygdalin, arbutin and sinigrin) are bitter sugars and turned out to activate hTAS2R16.

Other studies [4,6-8] focused on the ligand selectivity of hTAS2R16 and investigated receptor activation using one or more of the aforementioned compounds (salicin, helicin, amygdalin, arbutin and/or sinigrin), together with other bitter glycopyranosides (e.g. phenyl-β-D-glucopyranoside). Altogether, about 30 compounds have been identified to activate hTAS2R16.

In summary, although the ligand library of reference [5] is different from those of the other studies, in all cases salicin, helicin, amygdalin, arbutin and sinigrin were found to activate hTAS2R16.

Text S2. Analysis of the previous models of the hTAS2R16/salicin complex.

Previous studies [7-10] have suggested different models for hTAS2R16 bound to its agonist salicin (see Fig. 1 for the chemical structure of the ligand). Sakurai and coworkers [7-9] proposed two alternative binding poses (referred to as models A and B), in which the glucose unit is oriented towards the extracellular or the intracellular side, respectively. In comparison, our binding pose (see the main text and Fig. 5 *E-F*) is similar to model A (i.e. they share the same orientation of the ligand inside the binding pocket) and will be hereafter referred to as "glucose-out".

Sakurai and coworkers designed mutagenesis experiments of the residues identified as putatively involved in binding in their computational models (i.e. E86, N89, F93, W94, H181, F240 and I243) [7-9]. Based on the obtained mutagenesis data, they concluded that model B (in which the glucose unit is flipped 180 degrees compared to our “glucose-out” binding pose) was more likely than model A. Since the models in reference [7] are unfortunately not available and the corresponding computational protocol does not contain enough details to reproduce it (e.g. the sequence alignment employed is missing), we resorted to visual inspection of the images and to a careful analysis of the description of the models in the text of reference [7]. Within the limitations of this qualitative picture, we noticed that the mutagenesis data in reference [7] are not enough to distinguish univocally between models A and B. In particular:

(i) Mutations of $Q177^{5.39}$ (to N, E or A) did not affect significantly the EC_{50}, probably indicating that this residue does not interact directly with the ligand. Hence, Sakurai and coworkers discarded model A, where $Q177^{5.39}$ forms a H-bond with the "outwardly-oriented" glucose moiety. However, we found that these mutagenesis data are still compatible with our glucose-out binding pose, in which $Q177^{5.39}$ is most likely involved in shaping the binding cavity.

(ii) Mutations of $E86^{3.33}$ (to D and Q) showed decreased EC_{50} values for all the ligands tested. Sakurai and coworkers used this data to support model B, in which the hydroxymethyl group of the "inwardly-oriented" phenyl moiety of salicin is forming a H-bond with $E86^{3.33}$. Nonetheless, in our opinion the model B does not explain why the $E86^{3.33}$ mutations still affect the EC_{50} for arbutin and phenyl-β-D-glucopyranoside, even though both ligands lack the phenyl substituent putatively interacting with $E86^{3.33}$. Instead, this may be explained, at least in part, by the H-bonds between $E86^{3.33}$ and the glucose unit observed for all three ligands in our simulations of the glucose-out pose.

(iii) Other residues tested experimentally by Sakurai and coworkers ($N89^{3.36}$ and $H181^{5.43}$) do not clearly discriminate between the two ligand orientations, in our opinion, since they interact with the ligand in both models. The loss of receptor response upon $N89^{3.36}$ mutation is compatible with both model A (showing a H-bond with the hydroxymethyl substituent of the phenyl aglycon) and model B (displaying a H-bond with the hydroxymethyl group of the glucose unit). In our glucose-out simulations, $N89^{3.36}$ is interacting with the glucose (either its hydroxyl groups or the glycosidic oxygen), thus providing evidence in favor of model A. Similarly, the decrease in EC_{50} upon $H181^{5.43}$ mutation is consistent with the formation of H-bonds with the glucose unit in both models, though involving different glucose hydroxyl groups.

(iv) The final set of mutations included a group of hydrophobic residues located at the bottom of the binding cavity ($F93^{3.40}$, $F240^{6.52}$ and $I243^{6.55}$) and all showed a decrease in EC_{50}. According to Sakurai and coworkers, these experimental data support model B, in which these residues are forming hydrophobic interactions with the glucose moiety. However, in our opinion they are also consistent with model A, where these residues can form hydrophobic interactions with the phenyl aglycon.

Therefore, a critical reevaluation of the comparison between the computational models and the experimental data of Sakurai and coworkers [7-9] seems to indicate that the orientation of salicin in the binding site is far from clear. Moreover, additional experiments [6] reported after the publication of the model in reference [7] offer further elements to discriminate between the two possible ligand orientations. Indeed, the new mutagenesis data suggest new receptor-ligand interactions that are absent in the model B proposed by Sakurai and coworkers [7,9]. Below we comment on two representative data sets: mutations of $E262^{7.39}$ in the extracellular part of the binding cavity and mutations of $L59^{2.53}$ and $V265^{7.42}$ in the intracellular part.

(i) The E262D mutation decreased only slightly the maximum receptor activity with salicin, while E262A completely abolishes receptor activation [6]; this indicates that $E262^{7.39}$ is probably interacting with salicin. However, model B would place $E262^{7.39}$ approximately in front of the outwardly-oriented aglycon, which does not have any available H-bond donor to interact with $E262^{7.39}$, as the hydroxymethyl substituent is already engaged in a H-bond with $E86^{3.33}$. Therefore, no interaction between $E262^{7.39}$ and salicin can be formed in model B, at odds with the new experimental data [6].

(ii) Mutations of $L59^{2.53}$ and $V265^{7.42}$ decreased or abolished the receptor response, respectively, indicating that these two residues are likely to interact with salicin [6]. Since both residues are located in the bottom part of the binding cavity, in model B they would be placed near the "inwardly-oriented" glucose unit. Given the hydrophobic nature of these two residues, it is difficult to envision any interaction with glucose, in contrast with the experimental data.

Altogether, the model B proposed by Sakurai and coworkers does not seem to be fully consistent with the new experimental data in reference [6]. A possible reason that might explain, at least in part, this discrepancy is the use of a structural model of rhodopsin as template to generate the homology model of hTAS2R16 [7]. In order to further validate this hypothesis, we built a binding pose similar to model B. Here, the ligand is almost parallel to the receptor axis, but with different orientation compared to the "glucose-out" binding pose described in our main text. The glucose unit is buried inside the receptor while the aglycon is pointing toward the extracellular side (see the Fig below, in which panel A shows the "glucose-in" binding pose and panel B one of the two "glucose-out" poses described in the main text).

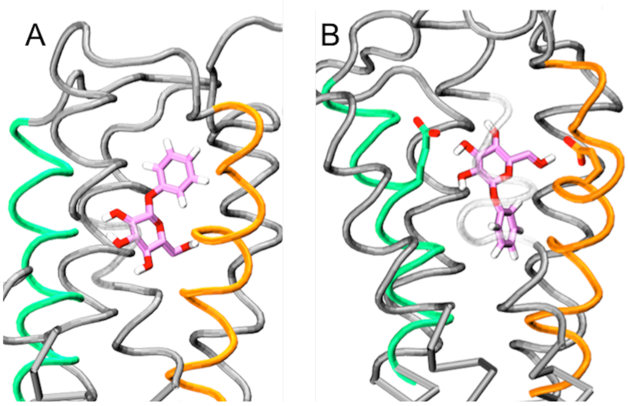

During the MM/CG simulation of this alternative "glucose-in" binding pose, we observed that the ligand already escaped from the binding site early in the simulation (see Figure S7). The instability of this pose is consistent with the absence of polar residues in the intracellular part of the binding cavity able to establish hydrogen bonds with the glucose unit and the lack of hydrophobic residues in the extracellular part that can interact with the aglycon moiety. Therefore, our simulations suggest that the "glucose-in" binding pose should be discarded.

More recently, Chen and coworkers performed molecular dynamics simulations of hTAS2R16 in complex with salicin (agonist) and probenecid (antagonist) to investigate the receptor activation mechanism [10]. However, the aim of their study was not focused on understanding the binding determinants of salicin and thus their comparison with the available experimental data was superficial. Upon visual inspection of the image in reference [10], corresponding to the initial docking pose, we noticed that the model of Chen and coworkers does not seem to be fully consistent with all the available experimental data [6,7]. Although some of the binding residues proposed by Chen and coworkers, such as $E86^{3.33}$ and $N89^{3.36}$, are compatible with the experimental data in reference [7], salicin appears to be too far away from $F93^{3.40}$, $F240^{6.52}$ and $I243^{6.55}$ to form any significant interaction with these residues. This is at odds with the mutagenesis data showing that mutations of these three residues decrease EC_{50} [7]. In addition, Chen and coworkers did not consider the experimental data in reference [6] when validating their proposed salicin binding pose. These new mutagenesis data suggest additional receptor-ligand interactions that are absent in the model proposed by Chen and coworkers [10]. Although they identified correctly $E262^{7.39}$ as one of the putative interacting residues, in their model salicin is not located deep enough inside the binding cavity to interact with $L59^{2.53}$, $F236^{6.48}$ and $V265^{7.42}$, at odds with the observation that mutations of these three residues decrease or abolish receptor response [6]. Therefore, the model of Chen and coworkers does not seem to be fully consistent with all the available experimental data [6,7].

Altogether, neither of the previously proposed models for the hTAS2R16/salicin seems to be fully compatible with all the available experimental data [6,7]. In an effort at clarifying the binding poses of the hTAS2R16 agonists complexes, in this work we have built structural models of hTAS2R16 in complex with three of its agonists (arbutin, salicin and phenyl-β-D-glucopyranoside) and submitted them to extensive molecular dynamics

simulations. The obtained computational models have been validated by comparison with the experimental data available as of December 2018 [6,7] (see Table S1).

Table S2. List of experimental mutagenesis data from reference [6,7] and calculation of precision and recall for the computational models of hTAS2R16 in complex with the three ligands considered in this work.

				PGP initial glucose-in		PGP initial glucose-out		PGP TM3		PGP TM7	
**Res**	**BW**	**EC$_{50}$ mut/EC$_{50}$ wt**	**PGP EC$_{50}$**	**Dist**	**Pred**	**Dist**	**Pred**	**Dist**	**Pred**	**Dist**	**Pred**
E86	3.33	D=5.1 Q=18.4	c	<	TP	<	FP	<	TP	<	TP
N89	3.36	Q,D,V,L,A=/	c	<	TP	<	FP	<	TP	<	TP
F93	3.40	L=14.2 Y=22.0 T=33.0 A=32.8	c	>	FN	>	FN	<	TP	<	TP
Q177	5.39	E=1.5 N=2.2 A=2.9	nc	>	TN	>	TN	<	FP	<	FP
H181	5.43	T=6.7 L=/	c	>	FN	>	FN	<	TP	<	TP
F240	6.52	W=6.5 Y=8.0 L=/	c	<	FP	>	FN	<	TP	>	FN
I243	6.55	V=4.1 A=/	c	<	FP	<	FP	>	FN	<	TP
				**PREC=** 0.50<br>**REC=** 0.50		**PREC=** 0.00<br>**REC=** 0.00		**PREC=** 0.83<br>**REC=** 0.83		**PREC=** 0.83<br>**REC=** 0.83	

				ARB TM3		ARB TM7	
**Res**	**BW**	**EC$_{50}$ mut/EC$_{50}$ wt**	**ARB EC$_{50}$**	**Dist**	**Pred**	**Dist**	**Pred**
E86	3.33	Q=2.9 D=7.9	c	<	TP	<	TP
N89	3.36	Q,D,V,L,A=/	c	<	TP	<	TP
F93	3.40	T,Y,L,A=/	c	<	TP	<	TP
Q177	5.39	E=0.9 A=1.6 N=1.7	nc	<	FP	<	FP
H181	5.43	T=2 L=/	c	<	TP	<	TP
F240	6.52	W=6.5 Y=8.0 L=/	c	>	FN	<	TP
I243	6.55	V=4.1 L=5.2 A=/	c	>	FN	>	FN
				**PREC=** 0.80<br>**REC=** 0.66		**PREC=** 0.83<br>**REC=** 0.83	

Res	BW	EC_{50} mut/EC_{50} wt	SAL EC_{50}	% of activity of wt	SAL TM3		SAL TM7	
					Dist	Pred	Dist	Pred
L59	2.53	No data		A=64				
E86	3.33	Q=5.7 D=9.8	c	No data	<	TP	<	TP
N89	3.36	Q,D,V,L,A=/	c	No data	<	TP	<	TP
F93	3.40	L=19.1 Y=45.9 A=52.8 T=55.1	c	No data	<	TP	<	TP
Q177	5.39	E=1.0 N=1.8 A=2.5	nc	No data	>	TN	>	TN
H181	5.43	T=8.5 L=59.3	c	No data	<	TP	<	TP
F236	6.48	No data		Y=2				
F240	6.52	W=9.4 Y=11.2 L=/	c	No data	>	FN	>	FN
I243	6.55	L=2.3 V=3.9 A=/	c	No data	>	FN	>	FN
E262		No data		D=92, A=0				
V265	7.42	No data		A=5				
					**PREC=** 1.00 **REC=** 0.66		**PREC=** 1.00 **REC=** 0.66	

The three ligands (LIG) are arbutin (ARB), salicin, (SAL) and phenyl-β-D-glucopyranoside (PGP). The first (Res) and second (BW) columns indicate the experimentally characterized hTAS2R16 residue [7] and its Ballesteros-Weinstein numbering [11], respectively. The ratio between mutant EC_{50} and wild type EC_{50} is reported in column three (EC_{50} mut/EC_{50} wt), followed by the fourth column (LIG EC_{50}) containing the interpretation of the EC_{50} values for the corresponding residue using the following nomenclature: c = change in EC_{50}; nc = no significant change in EC_{50}. "PGP initial" refers to the initial poses of phenyl-β-D-glucopyranoside, with the glucose unit either pointing inwards (glucose-in) or outwards (glucose-out). LIG TM3 and LIG TM7 correspond to the two binding modes (TM3-facing and TM7-facing, respectively) investigated in this work for the glucose-out orientation. For each binding mode, the "Dist" column indicates whether the corresponding residue is below or above (< or >, respectively) the distance threshold (5.5 Å) used to define the binding cavity, whereas the "Pred" column reports the test outcome for that residue (TP=true positive, TN=true negative, FP=false positive, FN=false negative), depending on the presence or absence of an actual chemical interaction. These predictions were used to calculate the statistical parameters precision (PREC) and recall (REC) (see main text and Fig. S3). The representative simulation snapshots used for this calculation were obtained following the procedure explained in the main text (see Methods section), except for "PGP initial". An additional column is included for salicin (% of activity of wt), summarizing the experimental data from [6].

Table S3. List of the simulations performed.

ligand	glucose orientation	TM facing mode
phenyl-β-D-glucopyranoside	in	–
	out	TM3
		TM7
arbutin	out	TM3
		TM7
salicin	out	TM3
		TM7

The glucose-out orientation refers to the binding pose described in the main text, while the glucose-in orientation (see above) corresponds to the ligand flipped vertically by 180 degrees with respect to the glucose-out one. In this binding pose, the glucose unit is pointing toward the intracellular side of the receptor and the aglycon sits in the extracellular part of the binding pocket. For the glucose-out orientation, two binding modes were explored (TM3- and TM7-facing, see the main text), which differ by a 180 degrees horizontal flip.

Figure S2. Sequence alignment. Input file for MODELLER.

```
>P1;tas2r16
sequence:sp_Q9NYV7_T2R16_HUMAN_Tas/1-291:   1: : 291: :: : 0.00: 0.00
MIPIQLTVFFMIIYVLESLTIIVQSSLIVAVLGREWLQVRRLMPVDMILISLGISRFCLQWASMLNNFCSYFNLN-----
YVLCNLTITWEFFNILTFWLNSLLTVFYCIKVSSFTHHIFLWLRWRILRLFPWILLGSLMITCVTIIPSAIGNYIQIQLL
TMEHLPRNSTVTDKLENFHQYQFQAHTVALVIPFILFLASTIFLMASLTKQIQHH---STGHCNPSMKARFTALRSLAVL
FIVFTSYFLTILITIIGTLFDKRCWLWVWEAFVYAFILMHSTSLMLSSPTLKRILKGKC*

>P1;4lde_A
structureX:4lde_A: :A: :A: : :2.79:
--DEVWVVGMGIVMSLIVLAIVFGNVLVITAIAK---FERLQTVTNYFITSLACADLVMGLAVVPFGAAHILTKTWTFGN
FWCEFWTSIDVLCVTASIETLCVIAVDRYFAITSPFKYQSLLTKNKARVIILMVWIVSGLTSFLPIQMHWYRATHQEA--
---INCYAEETCCDFFTNQAYAIASSIVSFYVPLVIMVFVYSRVFQEAKRQLQK-------FALKEHKALKTLGIIMGTF
TLCWLPFFIVNIVHVIQDNLIRKEVYILLNWIGYVNSGFNPLIYC-RSPDFRIAFQELL*
```

Text S3. Example of the calculation of precision and recall.

Precision and recall were calculated based on the data in Supplementary Tables S2 and S4-S7. Here we show as an example the values calculated for hTAS2R16 in complex with phenyl-β-D-glucopyranoside in the TM3 binding mode. In Supplementary Table S2, we first analyzed the experimental mutagenesis data. Experimental binding residues (EB) are those whose mutation causes an increase in EC_{50} higher than 5-fold compared to the wild-type (EC_{50} mut / EC_{50} wt > 5), while residues whose mutation does not change significantly the EC_{50} value ("nc" in the PGP EC_{50} column of Supplementary Table S2) are considered as experimental non binding residues (ENB). The results of this experimentally-based classification are shown below:

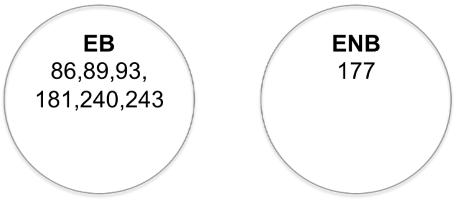

Then, we analyzed the data obtained from the MM/CG molecular dynamics simulation of the same complex (PGP TM3 column in Supplementary Table S2). A computational binding residue (CB) was defined according to three criteria:

1) distance within 5.5 Å from the closest ligand atom ("PGP TM3 Dist" column in Supplementary Table S2);

2) rationality of the chemical interaction;
3) persistency of the interaction.
For criterium (2), our hydrogen bond definition takes into account implicitly that the residue/ligand atoms involved in the interaction are within a distance of 3.5 Å (together with a 30° deviation for the angle made by donor, hydrogen and acceptor atoms). Instead, for residues forming hydrophobic interactions we applied a 5.5 Å residue/ligand distance cutoff. For criterium (3), we used two different persistency cutoff values. We considered a residue as CB when its hydrogen bond persistency with at least one hydroxyl group of the ligand is higher than 10% (Supplementary Table S4) or when its hydrophobic interaction persistency is at least 80% (Supplementary Table S6). The results of this computationally-based classification are shown below:

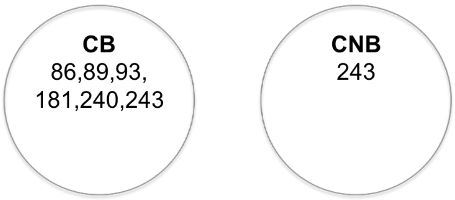

Next, a comparison between the experimental and computational binding and non binding residues was performed to classify the residues into 4 different outcomes. A residue that belongs to both CB and EB groups was considered as a true positive (TP), to CNB and ENB as a true negative (TN), to CB and ENB as a false positive (FP), and to CNB and EB as a false negative (FN) (see figure below and paragraph "Interaction analysis" in the main text).

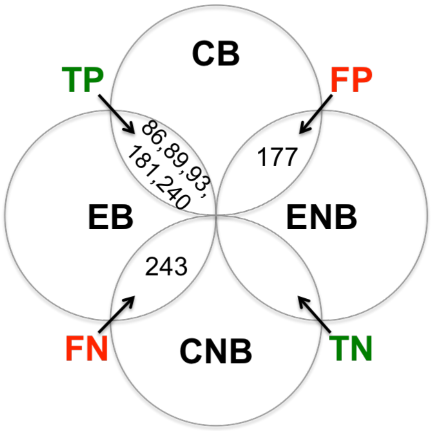

Finally, the total number of TP (five), FP (one) and FN (one) was counted to calculate precision and recall according to the equations presented in the main text. In this example, precision and recall are both 0.83 (Supplementary Table S2), indicating a good agreement of the computational model with the experimental data. For the analysis of the static binding poses (PGP initial glucose-out/-in in Supplementary Table S2), we used a similar protocol to calculate precision and recall, except for the definition of computational binding and non-binding residues. "Computational binding" and "computational non-binding" residues were defined based solely on the presence and absence, respectively, of protein/ligand interactions (criteria 1 and 2 in the text above) and did not include the persistency (criterium 3). This is because the interactions in the static binding poses are not time dependent, since no MD simulation had been run at this point.

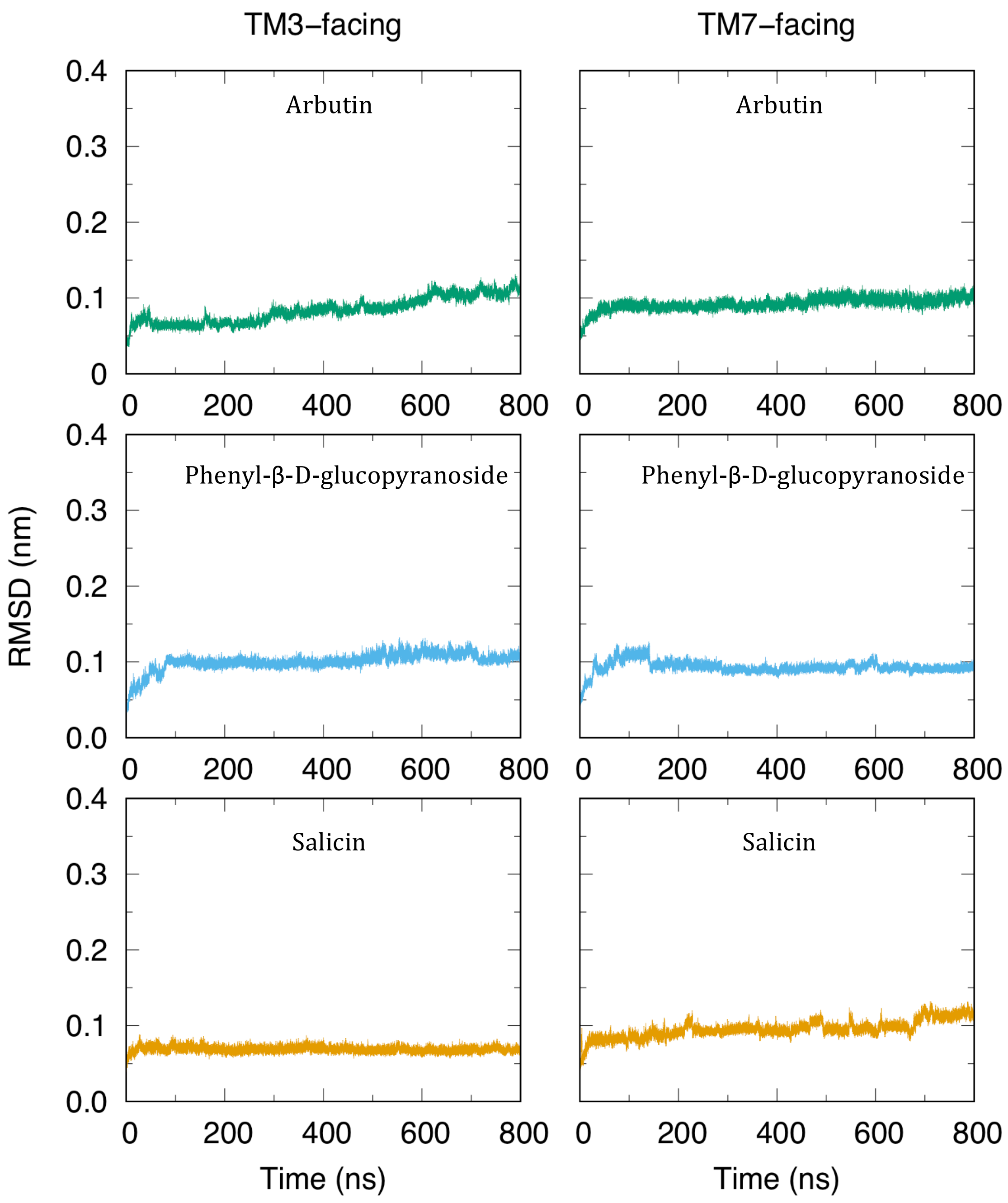

Figure S3. RMSD of the transmembrane helices plotted as a function of the simulation time. The RMSD was calculated including only the Cα atoms and using as reference the initial frame of each simulation.

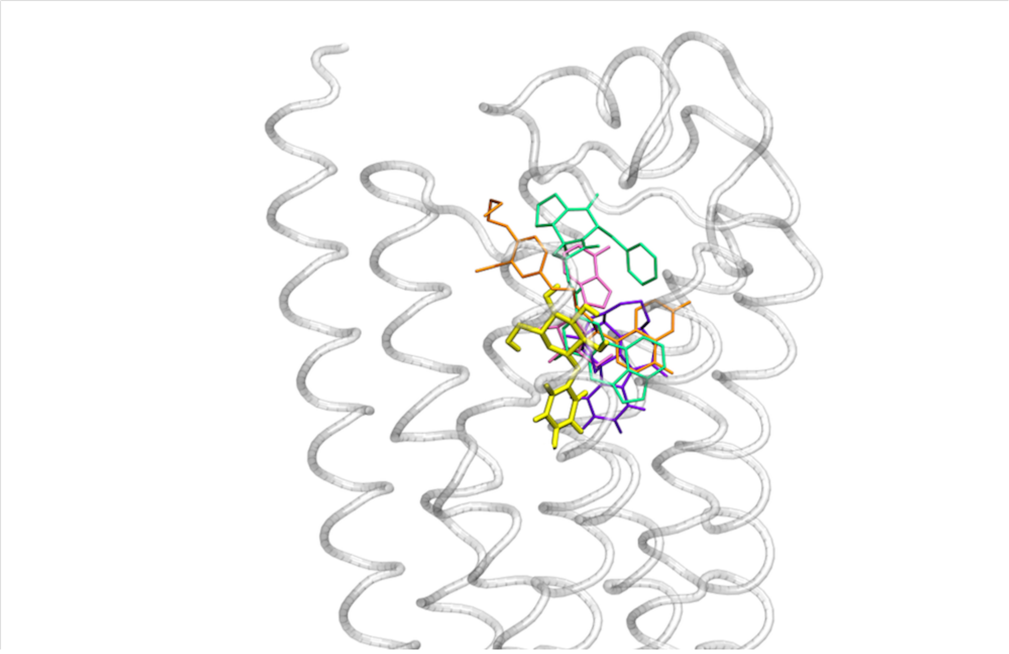

Figure S4. Structure of hTAS2R16 in complex with phenyl-β-D-glucopyranoside superimposed with the position of other GPCR agonists for which receptor/ligand crystal structures are available. Phenyl-β-D-glucopyranoside is in yellow, serotonin receptor 5-HT_1/ergotamine (PDB ID: 4IAR) in green, [12], β_2 adrenergic receptor/FAUC50 (PDB ID: 3PDS) in orange [13] and adenosine A_{2A} receptor/adenosine (PDB ID: 2YDO) in pink [14]. In addition, the position of strychnine bound to the bitter taste receptor hTAS2R46, [15] found using a similar computational approach as in this work, is shown in violet.

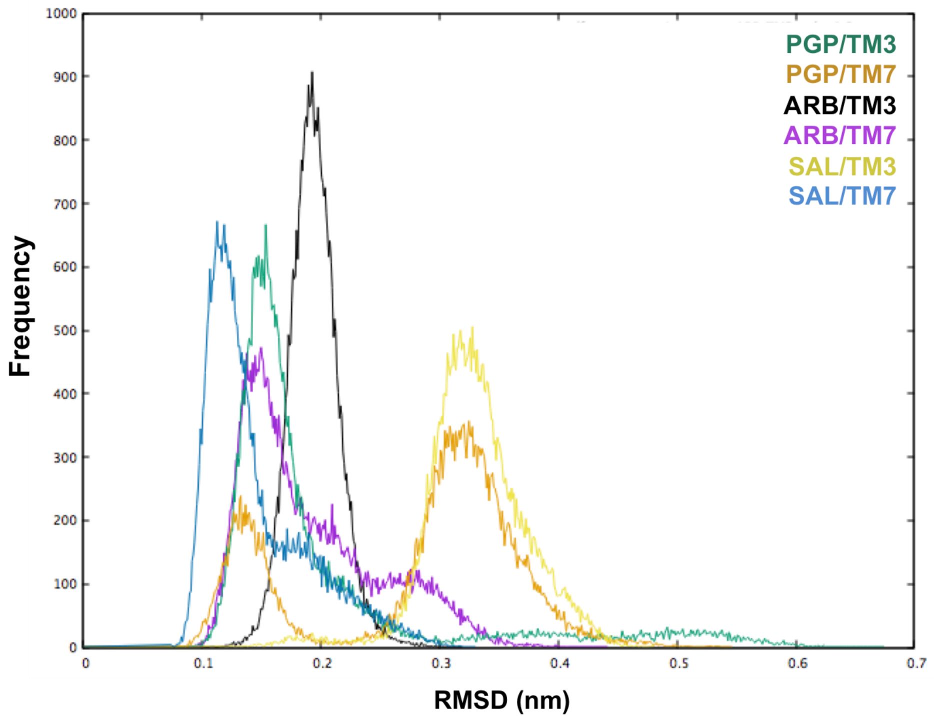

Figure S5. RMSD of the ligand heavy atoms represented as distribution. The initial frame of the corresponding simulation was used as reference structure. TM3 and TM7 indicate, respectively, the TM3 and TM7 ligand binding mode. We observed that two simulations (PGP TM7 and SAL TM3) display larger RMSD values. The presence of two peaks for the phenyl-β-glucopyranoside in TM7 facing binding mode (orange line) is due to a shift of the ligand that, after 200 ns, moves approximately by 2 Å. Phenyl-β-glucopyranoside is, among the three ligands studied, the only one without any hydroxyl group as a substituent of the phenyl group and, hence, it cannot form a hydrogen bond with T92$^{3.39}$, a residue positioned at the bottom part of the binding cavity. This missing interaction allows the ligand to fluctuate up and down, while still keeping the hydrophobic aglycon in contact with the bottom, hydrophobic region of the binding site (together with the glucose ring forming H-bonds with the polar and charged residues in the top part of the binding pocket). The shift of the RMSD frequency peak for the salicin TM3 binding mode (yellow line) is due to a rotation along the receptor axis of the hydrophobic moiety of the ligand at the beginning of the simulation, which allows the hydroxymethyl substituent of the phenyl group to bind T92.

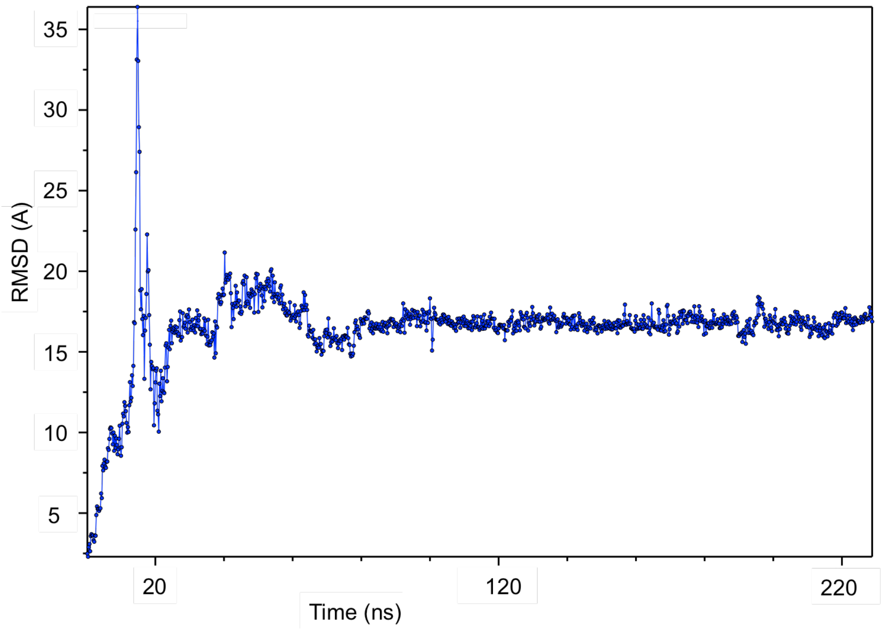

Figure S6. Ligand RMSD of the glucose-in binding pose. The ligand escapes from the binding site after few nanoseconds, moving into the extracellular space.

Table S4. Persistency of H-bonds between hTAS2R16 and the three agonists in the TM3-facing binding mode.

res	BW	res atom	lig atom	ARB direct (%)	ARB wm (%)	PGP direct (%)	PGP wm (%)	SAL direct (%)	SAL wm (%)
**S63**	2.57	OG	O2	<10	<10	<10	<10	26	10
			O3	<10	<10	<10	10	28	18
**N66**	2.60	ND2	O4	<10	<10	<10	<10	11	12
**N67**	2.61	OD1	O3	<10	19	<10	<10	<10	<10
**E86**	3.33	OE1	O4	<10	<10	<10	<10	29	<10
			O6	50	<10	36	<10	27	<10
		OE2	O4	<10	<10	<10	<10	27	<10
			O6	34	<10	44	<10	26	<10
**N89**	3.36	ND2	O1	<10	<10	<10	<10	16	<10
			O5	30	<10	<10	<10	44	<10
			O6	16	<10	45	<10	<10	<10
**T92**	3.39	OG1	O7	23	<10	<10	<10	<10	<10
			O	<10	<10	<10	<10	65	<10
**Y176**	5.38	OH	O4	<10	<10	<10	25	<10	<10
**Q177**	5.39	NE2	O4	50	<10	<10	13	<10	<10
**Y239**	6.51	OH	O6	<10	<10	<10	16	30	<10
**E262**	7.39	OE1	O2	<10	<10	18	<10	<10	<10
			O3	48	<10	<10	10	<10	<10
			O4	22	<10	<10	<10	10	14
		OE2	O2	<10	<10	13	<10	<10	<10
			O3	37	<10	<10	10	<10	<10
			O4	19	<10	<10	<10	10	13
**Y266**	7.43	OH	O2	70	<10	35	<10	<10	<10
			O3	<10	<10	<10	11	<10	<10

Columns 1 and 2 indicate the interacting receptor residue, together with its Ballesteros-Weinstein numbering. The atoms involved in H-bonds are specified in columns 3 (residue atom) and 4 (ligand atom). The H-bond persistency (expressed as a percentage of the total simulation) is shown in columns 5, 6 and 7 (direct H-bonds) and columns 6, 8 and 10 (water-mediated H-bonds). Only H-bonds with persistency larger than 10% are reported (colored in green). The darkness of the green font is related with the persistency of the H-bond, i.e. the darker the green, the greater the persistency.

Table S5. Persistency of H-bonds between hTAS2R16 and the three agonists in the TM7-facing binding mode.

res	BW	res atom	lig atom	ARB direct (%)	ARB wm (%)	PGP direct (%)	PGP wm (%)	SAL direct (%)	SAL wm (%)
**N67**	2.61	ND2	O6	<10	<10	<10	<10	**61**	<10
**E86**	3.33	OE1	O2	**55**	<10	**41**	<10	**27**	<10
			O3	**50**	<10	**26**	<10	**19**	<10
		OE2	O2	**36**	<10	**52**	<10	**30**	<10
			O3	**32**	<10	**27**	<10	**28**	<10
**N89**	3.36	ND2	O	<10	<10	<10	<10	**61**	<10
			O1	**48**	<10	**18**	<10	**10**	<10
			O2	**27**	<10	**18**	<10	<10	<10
**T92**	3.39	OG1	O7	**39**	<10	<10	<10	<10	<10
	5.39	NE2	O3	<10	**12**	**11**	<10	<10	<10
		OE1	O4	<10	<10	<10	**12**	<10	<10
**Y239**	6.51	OH	O5	<10	<10	**48**	<10	<10	<10
**E262**	7.39	OE1	O3	<10	**15**	<10	<10	**16**	**12**
			O4	<10	**11**	<10	<10	**26**	**12**
			O6	<10	<10	**30**	<10	<10	<10
		OE2	O3	<10	**16**	<10	<10	**16**	**13**
			O4	<10	**12**	<10	<10	**26**	**10**
			O6	<10	<10	**32**	<10	<10	<10
**Y266**	7.43	OH	O6	**55**	<10	**20**	<10	<10	<10

Columns 1 and 2 indicate the interacting receptor residue, together with its Ballesteros-Weinstein numbering. The atoms involved in H-bonds are specified in columns 3 (residue atom) and 4 (ligand atom). The H-bond persistency (expressed as a percentage of the total simulation) is shown in columns 5, 6 and 7 (direct H-bonds) and columns 6, 8 and 10 (water-mediated H-bonds). Only H-bonds with persistency larger than 10% are reported (colored in green). The darkness of the green font is related with the persistency of the H-bond, i.e. the darker the green, the greater the persistency.

Table S6. Percentage of time that each residue spends with at least an atom-atom contact with the ligand in the TM3-facing binding mode simulations.

Res	BW	ARB	PGP	SAL
L59	2.53	100	100	97
A62	2.56	<10	<10	21
S63	2.57	<10	27	99
M64	2.58	27	<10	<10
N66	2.60	<10	12	99
N67	2.61	97	48	21
T82	3.29	79	38	<10
W85	3.32	100	100	100
E86	3.33	100	95	100
F88	3.35	100	100	99
N89	3.36	100	97	100
I90	3.37	45	11	<10
T92	3.39	100	90	100
F93	3.40	100	90	100
K169	ECL2	88	<10	<10
L170	5.32	<10	35	<10
E171	5.33	29	<10	<10
N172	5.34	98	<10	<10

Res	BW	ARB	PGP	SAL
Y176	5.38	19	95	72
Q177	5.39	97	37	10
H181	5.43	100	95	92
V235	6.47	<10	<10	98
F236	6.48	100	95	100
Y239	6.51	100	100	100
P240	6.52	<10	55	23
L258	7.35	<10	60	<10
W259	7.36	50	<10	<10
W261	7.38	14	21	94
E262	7.39	100	100	100
A263	7.40	65	12	60
F264	7.41	22	<10	97
V265	7.42	100	100	100
Y266	7.43	100	100	28
F268	7.45	76	77	93
I269	7.46	95	86	<10

A contact is defined when this distance is below 5.5 Å. Contacts with percentage 90% or larger are highlighted in red, and those with percentage between 80% and 90% in orange.

Table S7. Percentage of time that each residue spends with at least an atom-atom contact with the ligand in the TM7-facing binding mode simulations.

Res	BW	ARB	PGP	SAL
**L59**	2.53	100	98	100
**S63**	2.57	40	<10	<10
**M64**	2.58	<10	<10	94
**N66**	2.60	27	34	13
**N67**	2.61	42	<10	100
**Y71**	2.65	<10	<10	19
**T82**	3.29	83	50	<10
**W85**	3.32	100	100	100
**E86**	3.33	100	100	100
**F88**	3.35	100	100	100
**N89**	3.36	100	100	100
**I90**	3.37	<10	<10	15
**T92**	3.39	100	83	100
**F93**	3.40	81	54	100
**T40**	3.41	<10	19	<10
**K169**	ECL2	<10	18	89
**L170**	5.32	<10	64	<10
**E171**	5.33	<10	37	<10
**N172**	5.34	24	31	<10
**F173**	5.35	20	<10	<10
**H174**	5.36	<10	53	<10
**Y176**	5.38	44	<10	<10
**Q177**	5.39	45	94	61

Res	BW	ARB	PGP	SAL
**F178**	5.40	<10	86	<10
**H181**	5.43	100	100	100
**V235**	6.47	30	<10	19
**F236**	6.48	100	85	100
**Y239**	6.51	100	100	100
**P240**	6.52	16	<10	28
**I243**	6.55	<10	59	<10
**I247**	6.59	<10	15	<10
**L251**	7.27	<10	21	<10
**F252**	7.28	<10	17	<10
**R255**	7.31	15	50	<10
**C256**	7.32	<10	<10	24
**L258**	7.35	<10	73	<10
**W259**	7.36	40	23	26
**W261**	7.38	<10	32	<10
**E262**	7.39	100	100	100
**A263**	7.40	94	52	100
**F264**	7.41	28	<10	12
**V265**	7.42	100	100	100
**Y266**	7.43	100	100	49
**F268**	7.45	94	20	82
**I269**	7.46	100	26	92

A contact is defined when this distance is below 5.5 Å. Contacts with percentage 90% or larger are highlighted in red, and those with percentage between 80% and 90% in orange.

Table S8. Residue conservation among hTAS2Rs.

Residue	Position	Conservation across the hTAS2R family (%)
R56	2.50	R 96, T 4
L59	2.53	L 92, M 4, Q 4
S63	2.57	I 40, L 28, M 16, F 4, G 4, S 4, V 4
N66	2.60	N 32, D 24, H 12, S 12, E 8, K 4, L 4, V 4
N67	2.61	W 40, N 16, G 12, S 8, T 8, A 4, C 4, I 4, L 4
W85	3.32	W 84, F 8, L 4, T 4
E86	3.33	A 28, T 20, V 16, H 8, I 8, M 8, D 4, E 4, L 4
N89	3.36	N 84, G 8, S 4, D 4
T92	3.39	S 64, N 16, T 16, E 4
F93	3.40	L 32, M 16, N 16, I 12, F 8, V 8, S 4, T 4
H181	5.43	T 28, L 24, F 12, K 8, A 4, C 4, G 4, H 4, M 4, S 3, Y 4
F189	5.51	F 72, L 24, T 4
F192	5.54	F 36, S 24, T 24, C 8, A 4, N 4
V235	6.47	L 84, F 12, V 4
F236	6.48	F 36, Y 28, C 20, L 8, H 4, R 4
Y239	6.51	Y 64, F 12, H 8, N 8, S 8
F240	6.52	F 60, S 12, Y 12, A 8, C 4, I 4
E262	7.39	E 32, Q 28, K 16, M 8, D 4, L 4, I 4, V 4
Y266	7.43	A 20, F 20, I 12, Y 12, M 8, N 8, V 8, G 4, L 4, T 4
F268	7.45	Y 68, C 16, F 12, S 4

The hTAS2R16 residue number is specified in the first column, the corresponding position in the hTAS2R family is in the second column (using the Ballesteros-Weinstein numbering [11]) and the conservation percentage for each of the residues found in that position is indicated in the last column.

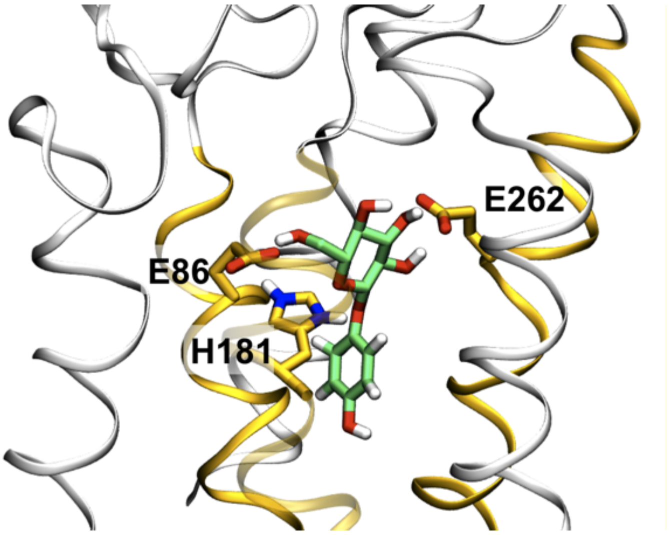

Figure S7. Interaction of E86$^{3.33}$ and E262$^{7.39}$ with the ligand and between H181$^{5.43}$ with E86$^{3.33}$. E86$^{3.33}$ and E262$^{7.39}$ form hydrogen bonds with the hydroxyl groups of the glucose unit, while H181$^{5.43}$ forms a stable salt bridge with E86$^{3.33}$ and thus has a fundamental role in keeping E86 in the right position to interact with the agonists.

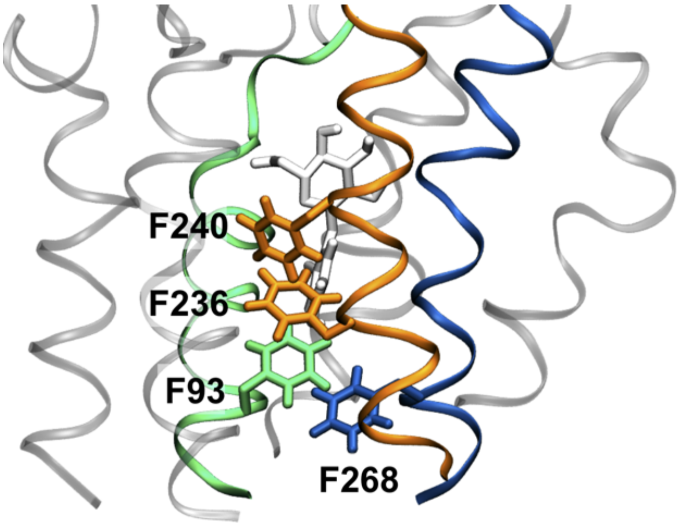

Figure S8. The phenylalanine cluster. It is formed by F93$^{3.40}$, F240$^{6.52}$, F236$^{6.48}$ and F268$^{7.45}$. F93$^{3.40}$, F236$^{6.48}$ and F268$^{7.45}$ are involved in hydrophobic interactions with the ligand, as well as forming π-π stacking interactions among them. Instead, F240$^{6.52}$ forms a T-stacking interaction with F236$^{6.48}$, and thus may help to stabilize the other phenylalanines in the cluster. The helices involved in the phenylalanine cluster are colored in green (TM3), orange (TM6) and blue (TM7).

Text S4. Speculation about possible alternative binding modes.

The simulations presented in the main text, in combination with the available experimental data, support the existence of two different binding modes in hTAS2R16 (TM3- and TM7-facing), in which the agonist is rotated by 180 degrees. Considering the bundle shape of the binding cavity, one could wonder whether a rotation by 90 degrees could enable other alternative binding modes (i.e. multiple binding mode mechanism).

If the ligands were rotated by 90 degrees with respect to the binding poses identified in the manuscript (TM3- and TM7-facing modes), the hydroxyl groups of the glucose ring would point towards TM2 on one side and TM5 and/or TM6 on the other side (hereafter "TM2-facing" and "TM5/6-facing" mode). This is at odds with the extensive mutagenesis and functional data in references [6,7]. Indeed, the authors of reference (Thomas et al. 2017) already proposed that the hTAS2R16 residues key for receptor specificity and activation are located on TM3 and TM7.

Since the aglycon is surrounded by hydrophobic residues (see Fig. 4 in the main text) and hydrophobic interactions are non-directional, we expect that the most significantly affected interactions upon a 90 degrees rotation are those with the glucose ring. As shown in the figure below, the polar residues in TM2, TM5 and TM6 that could potentially interact with glucose would be S63 and N66 on TM2, H181 on TM5 and Y239 on TM6 (note that we do not consider Q177 on TM5 because site-directed mutagenesis data [7] indicates that this residue is not involved in ligand binding). Compared to the glucose binding residues in the TM3- and TM7-facing modes (E86 and N89 in TM3 and E262 and Y266 in TM7, see Fig. 3 main text), there are two equivalent (N and Y) and two different residues (S and H, instead of two E). S63 and H181 can form only one H-bond each, whereas each of the E86 and E262 residues can establish two H-bonds. Therefore, it is reasonable to think that the H-bond networks in the TM3 and TM7-facing modes are stronger than in the TM2 and TM5/6 modes. In addition, the TM3 and TM7 helices are much closer than TM2 to TM5/6. The distance between the Cα atoms of the mirroring residues E86 on TM3 and E262 on TM7 is ~12.7 Å, whereas that between the counterparts N66 on TM2 and H181 on TM5 is ~20.0 Å. As a consequence, we expect that the ligand would be more tightly bound in the TM3 and TM7-facing modes.

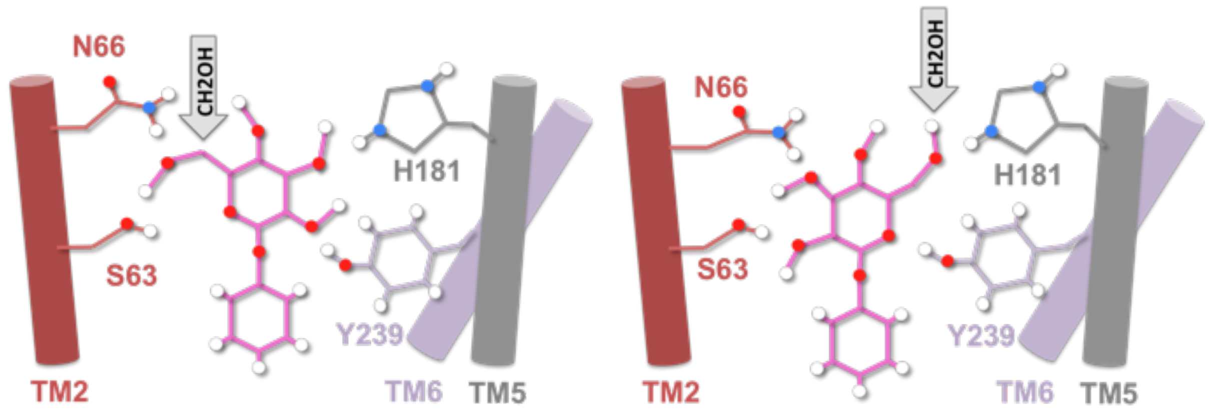

Altogether, the glucose/protein stabilizing interactions in the TM2 and TM5/6-facing modes are expected to be weaker than in the TM3 and TM7-facing modes. In addition, the TM2- and TM5/6-facing modes appear not to offer a molecular explanation for (i) the complete loss of receptor activity upon E262A mutation and (ii) the significant EC50 changes observed for isosteric (E86Q) or conservative (E86D) mutations. Therefore, the TM2- and TM5/6-facing modes are unlikely to contribute to ligand binding and we expect that the corresponding MD simulations would show that these two binding poses are significantly less stable.

Supporting references

1 Wiener, A., Shudler, M., Levit, A. & Niv, M. Y. BitterDB: a database of bitter compounds. *Nucleic Acids Res* **40**, D413-D419, doi:10.1093/nar/gkr755 (2012).

2 Bachmanov, A. A. & Beauchamp, G. K. Taste receptor genes. *Annu. Rev. Nutr.* **27**, 389-414 (2007).

3 Meyerhof, W. Elucidation of mammalian bitter taste. *Rev Physiol Bioch P* **154**, 37-72, doi:10.1007/s10254-005-0041-0 (2005).

4 Bufe, B., Hofmann, T., Krautwurst, D., Raguse, J. D. & Meyerhof, W. The human TAS2R16 receptor mediates bitter taste in response to beta-glucopyranosides. *Nat Genet* **32**, 397-401, doi:10.1038/ng1014 (2002).

5 Meyerhof, W. *et al.* The molecular receptive ranges of human TAS2R bitter taste receptors. *Chem Senses* **35**, 157-170, doi:10.1093/chemse/bjp092 (2010).

6 Thomas, A. *et al.* The bitter taste receptor TAS2R16 achieves high specificity and accommodates diverse glycoside ligands by using a two-faced binding pocket. *Sci Rep-Uk* **7**, 7753 (2017).

7 Sakurai, T. *et al.* Characterization of the beta-D-glucopyranoside binding site of the human bitter taste receptor hTAS2R16. *J Biol Chem* **285**, 28373-28378, doi:10.1074/jbc.M110.144444 (2010).

8 Sakurai, T. *et al.* The human bitter taste receptor, hTAS2R16, discriminates slight differences in the configuration of disaccharides. *Biochem Biophys Res Commun* **402**, 595-601, doi:10.1016/j.bbrc.2010.10.059 (2010).

9 Imai, H. *et al.* Amino acid residues of bitter taste receptor TAS2R16 that determine sensitivity in primates to beta-glycosides. *Biophys Physicobiol* **13**, 165-171, doi:10.2142/biophysico.13.0_165 (2016).

10 Chen, Z. *et al.* Insights into the binding of agonist and antagonist to TAS2R16 receptor: a molecular simulation study. *Molecular Simulation* **44**, 322-329 (2018).

11 Ballesteros, J. A. & Weinstein, H. [19] Integrated methods for the construction of three-dimensional models and computational probing of structure-function relations in G protein-coupled receptors. *Methods in neurosciences* **25**, 366-428 (1995).

12 Wang, C. *et al.* Structural Basis for Molecular Recognition at Serotonin Receptors. *Science* **340**, 610-614, doi:10.1126/science.1232807 (2013).

13 Rosenbaum, D. M. *et al.* Structure and function of an irreversible agonist-beta(2) adrenoceptor complex. *Nature* **469**, 236-240, doi:10.1038/nature09665 (2011).

14 Lebon, G., Bennett, K., Jazayeri, A. & Tate, C. G. Thermostabilisation of an agonist-bound conformation of the human adenosine A(2A) receptor. *J Mol Biol* **409**, 298-310, doi:10.1016/j.jmb.2011.03.075 (2011).

15 Sandal, M. *et al.* Evidence for a Transient Additional Ligand Binding Site in the TAS2R46 Bitter Taste Receptor. *J Chem Theory Comput* **11**, 4439-4449, doi:10.1021/acs.jctc.5b00472 (2015).
